# Elucidating Mitochondrial DNA Markers of *Ogura*-Based CMS Lines in Indian Cauliflowers (*Brassica oleracea* var. *botrytis* L.) and Their Floral Abnormalities Due to Diversity in Cytonuclear Interactions

**DOI:** 10.3389/fpls.2021.631489

**Published:** 2021-04-30

**Authors:** Saurabh Singh, Reeta Bhatia, Raj Kumar, Tusar K. Behera, Khushboo Kumari, Achintya Pramanik, Hemant Ghemeray, Kanika Sharma, R. C. Bhattacharya, Shyam S. Dey

**Affiliations:** ^1^Division of Vegetable Science, ICAR-Indian Agricultural Research Institute, New Delhi, India; ^2^Division of Floriculture and Landscaping, ICAR-Indian Agricultural Research Institute, New Delhi, India; ^3^ICAR-Indian Agricultural Research Institute, Regional Station, Kullu Valley, India; ^4^ICAR-National Institute for Plant Biotechnology, New Delhi, India

**Keywords:** *Brassica oleracea*, cytoplasmic male sterility, mitotype-specific markers, sequence analysis, cytonuclear interactions, floral malformations, floral reproductive traits

## Abstract

Mitochondrial markers can be used to differentiate diverse mitotypes as well as cytoplasms in angiosperms. In cauliflower, cultivation of hybrids is pivotal in remunerative agriculture and cytoplasmic male sterile lines constitute an important component of the hybrid breeding. In diversifying the source of male sterility, it is essential to appropriately differentiate among the available male sterile cytoplasms in cauliflower. PCR polymorphism at the key mitochondrial genes associated with male sterility will be instrumental in analyzing, molecular characterization, and development of mitotype-specific markers for differentiation of different cytoplasmic sources. Presence of auto- and alloplasmic cytonuclear combinations result in complex floral abnormalities. In this context, the present investigation highlighted the utility of organelle genome-based markers in distinguishing cytoplasm types in Indian cauliflowers and unveils the epistatic effects of the cytonuclear interactions influencing floral phenotypes. In PCR-based analysis using a set of primers targeted to *orf-138*, 76 Indian cauliflower lines depicted the presence of *Ogura* cytoplasm albeit the amplicons generated exhibited polymorphism within the *ofr-138* sequence. The polymorphic fragments were found to be spanning over 200–280 bp and 410–470 bp genomic regions of *BnTR4* and *orf125*, respectively. Sequence analysis revealed that such cytoplasmic genetic variations could be attributed to single nucleotide polymorphisms and insertion or deletions of 31/51 nucleotides. The cytoplasmic effects on varying nuclear-genetic backgrounds rendered an array of floral abnormalities like reduction in flower size, fused flowers, splitted style with the exposed ovule, absence of nonfunctional stamens, and petaloid stamens. These floral malformations caused dysplasia of flower structure affecting female fertility with inefficient nectar production. The finding provides an important reference to ameliorate understanding of mechanism of cytonuclear interactions in floral organ development in *Brassicas.* The study paves the way for unraveling developmental biology of CMS phenotypes in eukaryotic organisms and intergenomic conflict in plant speciation.

## Introduction

Mitochondria play a central role in energy production by oxidative phosphorylation, apoptosis, and other cellular processes (Chen et al., 2017; Shu et al., 2018). The migration of essential genes from mitochondrial genome to nuclear genome has been a consistent feature of genome evolution in eukaryotic organisms (Christensen, 2013; Horn et al., 2014). The integrated action of mitochondrial and nuclear genomes is pivotal for many of the cellular functions. The substantial structural complexity and variation in genome size of plant mitochondrial genome is resulted from the accumulation of diverse repeat sequences, frequent homologous recombination of large repeat sequences, and inclusion of foreign DNA fragments (Sloan, 2013; Gualberto and Kuhn, 2014; Gualberto et al., 2014; Chen et al., 2017). The plant mitogenomes also have the remarkable features of presence of highly diverse intergenetic regions, frequent mt-DNA rearrangements, slow evolution in mt-DNA sequences, and rapid structural evolution (Sloan, 2013; Chen et al., 2017). Attributed to these characteristics, the extensive genome reorganization and shuffling in gene order may occur in plant mitogenomes and unusual open reading frames (*ORFs*) are generated, some of which causes extreme phenotypes such as cytoplasmic male sterility (CMS) (Tanaka et al., 2012; Chen et al., 2017; Singh et al., 2019a). The CMS is a maternally inherited trait which is characterized by inability to produce viable pollen or male reproductive organs for sexual fertilization (Singh et al., 2019a). The mitochondrial genes determining CMS phenotype can be masked by fertility restorer genes (*Rf*) in nuclear genome. Presence of male sterile cytoplasm without the *Rf* gene in the nuclear genome results in CMS phenotype (Chen et al., 2017; Singh et al., 2019a). CMS is an important trait to provide new insights into plant nucleo-mitochondrial communication.

Cytoplasm-induced male sterility is a widespread phenomenon reported in over 150 plant species (Chase, 2007; Bohra et al., 2016; Chen et al., 2017). The CMS system is instrumental for utilization of heterosis in vegetable *Brassicas* for higher yield and quality traits (Dey et al., 2011a, 2018; Thakur et al., 2015; Singh et al., 2018a, 2019b,c). Cauliflower (*Brassica oleracea* var. *botrytis* L.) is an important member of vegetable *Brassicas* grown worldwide. It has been an important contributor in human diet owing to high content of dietary minerals, vitamins, glucosinolates, and other phytochemical compounds with antioxidant and anticancer properties (Singh et al., 2018a,b, 2019b). Availability of genetic mechanisms like sporophytic self-incompatibility (SSI) and CMS make the hybrid breeding in cauliflower remunerative (Dey et al., 2014; Sehgal and Singh, 2018; Singh et al., 2019b,c). However, nonavailability of strong S-alleles for reliable SSI system and instability of the S-alleles under varied climatic conditions made this system less attractive. Therefore, presently CMS is most widely used in hybrid breeding in cauliflower (Dey et al., 2013, 2014; Singh et al., 2021). The wild allies and other closely related *Brassica* coenospecies serve as the reservoir of different cytoplasms which are being used to develop CMS through intraspecific/interspecific/intergeneric hybridization mediated by sexual crossing or protoplast fusion (Cardi and Earle, 1997; Yamagishi and Bhat, 2014; Shu et al., 2016; Kang et al., 2017; Singh et al., 2019a; Bhatia et al., 2021a,b). Among the different CMS types, *ogu* (Ogura, 1968) and *pol* (Handa et al., 1995) CMS systems are widely used in hybrid breeding of vegetable *Brassicas*. Numerous CMS-related genes (*orfs*) have been identified and well characterized in different plant species. The key mitochondrial genes (*orfs*) associated with different CMS types in *Brassicaceae* are *orf138* correlated with *Ogura* CMS (Ogura, 1968; Singh et al., 2019a), *orf125* associated with *kosena* CMS of Radish (Iwabuchi et al., 1999), *orf72* correlated with *mur* CMS of *Diplotaxis muralis* (Shinada et al., 2006), *orf222* associated with “*nap CMS*” of *Brassica napus* (L’Homme et al., 1997), *orf224/atp6* correlated with *pol* CMS (Wang et al., 1995), *orf288* linked with *hau* CMS of *B. juncea* (Heng et al., 2018), *orf263* correlated with “*tour CMS*” of *Brassica tournefortii* (Landgren et al., 1996), and *orf220* associated with “tuber mustard CMS” of *Brassica juncea* (Yang et al., 2010). The gene-specific primers for these mitochondrial genomic regions can be efficiently utilized to differentiate different CMS types in *Brassica* crops. Several mitochondrial DNA-specific microsatellites (mt-SSR) markers have been developed to distinguish different CMS types and assessment of cytoplasmic diversity in different *Brassica* species like *B. oleracea* var. *italic* (Shu et al., 2015, 2016), *B. oleracea* var. *capitata* (Wang et al., 2012), *B. rapa* (Zamani-Nour et al., 2013; Zhang et al., 2013; Heng et al., 2015), *B. napus*, and *B. juncea* (Yu et al., 2014).

The CMS cauliflowers and other *Brassica* crops with *Ogura* or other alien cytoplasts display complex variations in reproductive features. These reproductive phenotypes are expressed as floral abnormalities such as homeotic-like transformation of stamens to pistil-like structures (pistillody), homeotic modification of stamens to petal-like structures (petaloidy), carpelloid stamens, adherence of functional stamens to style, partially opened flowers, splitted style with exposed ovule, splitted style, absence of nectaries, rudimentary nectaries, and fused flowers (Meur et al., 2006; Saha et al., 2016; Dey et al., 2017b; Kang et al., 2017; Singh et al., 2019a). These floral malformations are a result of incongruity between nuclear and mitochondrial genomes (Shu et al., 2015; Singh et al., 2019a). It is evident that loss of function, mutation, or insertion/deletion in MADS-box genes *APETALA2*, *APETALA3*, or *PISTILLATA*, which are class B genes of classic ABC model for flower development, renders homeotic transformation of stamens to carpels or petals to sepals (Zhang et al., 2011, 2018; Shu et al., 2015). The homeotic floral deformities have also been explained by the dosage imbalance of class B and class C genes of classic ABC model and aberrant mitochondrial gene expression (Meur et al., 2006; Liu et al., 2018). These floral malformations which are linked to genetic background and cytoplasm types, results in inefficient nectar production, impaired pollination, reduced female fertility, and consequently a drastic reduction in seed yield. The other alterations in flower phenotype and reproductive structures, such as aborted pollen, degenerative anthers, crooked or bent stigma, reduction in flower size, variation in filament size, etc., are universal in autoplasmic and alloplasmic CMS systems of *Brassica* crops (Shu et al., 2016; Dey et al., 2017a; Kang et al., 2017). In India, during the last three decades, a large number of CMS-based breeding lines/material have been generated in Indian cauliflowers by repeated backcrossing or somatic hybridization by exploiting various CMS sources. However, systematic characterization and identification of these floral malformations in CMS lines of the Indian cauliflowers have not been done so far.

Furthermore, mitochondrial markers can potentially be used to determine the CMS mitotypes and analyze genetic diversity in Indian cauliflower CMS lines. Moreover, mitotype-specific SSRs can be reliably used for distinguishing normal CMS lines from the one having varying degree of floral deformities. Identification of mt-SSRs and other mitochondria-specific markers associated with floral deformities will enable to screen out cauliflower CMS lines with different types of floral abnormalities at an early stage. Analysis of sequence-based variation and its association with floral reproductive development will be instrumental in elucidating developmental biology of floral organ development in CMS phenotypes of eukaryotic organisms.

## Materials and Methods

### Plant Materials and Nucleic Acid Isolation

The basic plant material consisted of 76 CMS accessions of cauliflower with different nuclear backgrounds, developed after more than nine generations of backcrossing (Table 1). Among the 76 genotypes, 63 CMS lines were developed at ICAR-Indian Agricultural Research Institute (IARI), Regional Station, Katrain, Kullu Valley, Himachal Pradesh, India through backcross introgression. Thirteen CMS based F1 hybrids were obtained from different private companies (Table 1). In addition, one fertile inbred line of cauliflower with normal cytoplasm, Sel-27 was used as control. The CMS lines along with control Sel-27 were grown in pro-trays under glasshouse conditions of Baragram Experimental Farm of ICAR-Indian Agricultural Research Institute (IARI), Regional Station, Katrain, Kullu Valley, Himachal Pradesh, India for extraction of nucleic acid. The deoxyribonucleic acid was isolated from fully expanding leaves (100 mg) from 25 to 30 days old seedlings using cetyltrimethyl ammonium bromide (CTAB) method with minor modifications (Murray and Thompson, 1980). The deoxyribonucleic acid samples were adjusted to 25–50 ng DNA/μl and were stored at −80°C until use.

**TABLE 1:** Large genetic stock of Indian cauliflower under study.

**Sr. No.**	**CMS accessions**	**Status**	**Backcross generations**	**Source**	**Leafiness/riceyness**
A1	Ogu122-5A	CMS line	BC_12_	IARI Katrain	Absent
A2	Ogu115-33A	CMS line	BC_9_	IARI Katrain	Absent
A3	Ogu118-6A	CMS line	BC_12_	IARI Katrain	Absent
A4	Ogu307-33A	CMS line	BC_9_	IARI Katrain	Absent
A5	Ogu33-1A	CMS line	BC_9_	IARI Katrain	Absent
A6	Ogu1A	CMS line	BC_9_	IARI Katrain	Absent
A7	Ogu309-2A	CMS line	BC_13_	IARI Katrain	Absent
A8	OguKt-2-6A	CMS line	BC_12_	IARI Katrain	Absent
A9	Ogu13-85-6A	CMS line	BC_12_	IARI Katrain	Absent
A10	Ogu16A	CMS line	BC_9_	IARI Katrain	Absent
A11	Ogu3A	CMS line	BC_9_	IARI Katrain	Absent
A12	Ogu2-6A	CMS line	BC_11_	IARI Katrain	Absent
A13	Ogu2A	CMS line	BC_9_	IARI Katrain	Absent
A14	Ogu14A	CMS line	BC_9_	IARI Katrain	Absent
A15	Ogu122-1A	CMS line	BC_13_	IARI Katrain	Absent
A16	OguKt-9-2A	CMS line	BC_13_	IARI Katrain	Absent
A17	Ogu121-1A	CMS line	BC_12_	IARI Katrain	Absent
A18	Ogu126-1A	CMS line	BC_13_	IARI Katrain	Absent
A19	Ogu134-8A	CMS line	BC_12_	IARI Katrain	Absent
A20	Ogu12A	CMS line	BC_9_	IARI Katrain	Absent
A21	Ogu119-1A	CMS line	BC_13_	IARI Katrain	Absent
A22	Ogu34A	CMS line	BC_9_	IARI Katrain	Absent
A23	Ogu178-8A	CMS line	BC_12_	IARI Katrain	Absent
A24	Ogu118-2A	CMS line	BC_11_	IARI Katrain	Absent
A25	Ogu33A	CMS line	BC_9_	IARI Katrain	Absent
A26	HVCF-29	Hybrid	–	AcsenHyVeg	Absent
A27	HVCF-18	Hybrid	–	AcsenHyVeg	Absent
A28	HVCF-16	Hybrid	–	AcsenHyVeg	Absent
A29	OguKt-2-1A	CMS line	BC_12_	IARI Katrain	Absent
A30	Ogu309-1A	CMS line	BC_11_	IARI Katrain	Absent
A31	Ogu-HL-1A	CMS line	BC_11_	IARI Katrain	Absent
A32	Ogu307-1A	CMS line	BC_12_	IARI Katrain	Absent
A33	Ogu Kt-8-2A	CMS line	BC_12_	IARI Katrain	Absent
A34	Ogu119-2A	CMS line	BC_11_	IARI Katrain	Absent
A35	Ogu121-2A	CMS line	BC_11_	IARI Katrain	Absent
A36	Ogu-HL-3A	CMS line	BC_11_	IARI Katrain	Absent
A37	Ogu13-85-3A	CMS line	BC_11_	IARI Katrain	Absent
A38	Ogu119-6A	CMS line	BC_12_	IARI Katrain	Absent
A39	Snowpearl	Hybrid	**–**	Syngenta	Absent
A40	CFH-1522	Hybrid	**–**	Syngenta	Absent
A41	Kimaya	Hybrid	**–**	Syngenta	Absent
A42	Pahuja	Hybrid	**–**	Pahuja seeds	Absent
A43	Ogu13A	CMS line	BC_9_	IARI Katrain	Absent
A44	Ogu34-1A	CMS line	BC_9_	IARI Katrain	Absent
A45	Ogu1-2A	CMS line	BC_12_	IARI Katrain	Absent
A46	Ogu13-85-2A	CMS line	BC_11_	IARI Katrain	Absent
A47	Ogu118-3A	CMS line	BC_12_	IARI Katrain	Absent
A48	Ogu307-6A	CMS line	BC_12_	IARI Katrain	Absent
A49	Ogu-HL-6A	CMS line	BC_11_	IARI Katrain	Absent
A50	Ogu308-6A	CMS line	BC_12_	IARI Katrain	Absent
A51	Ogu13-01-5A	CMS line	BC_9_	IARI Katrain	Absent
A52	Ogu13-85-4A	CMS line	BC_11_	IARI Katrain	Absent
A53	Ogu118-4A	CMS line	BC_12_	IARI Katrain	Absent
A54	Ogu76-4A	CMS line	BC_9_	IARI Katrain	Absent
A55	Ogu33-4A	CMS line	BC_9_	IARI Katrain	Absent
A56	Ogu77-4A	CMS line	BC_12_	IARI Katrain	Absent
A57	Ogu13-85-33A	CMS line	BC_11_	IARI Katrain	Absent
A58	Ogu76-33A	CMS line	BC_11_	IARI Katrain	Absent
A59	Ogu122-8A	CMS line	BC_12_	IARI Katrain	Absent
A60	Ogu1-8A	CMS line	BC_12_	IARI Katrain	Absent
A61	Ogu309-8A	CMS line	BC_13_	IARI Katrain	Absent
A62	Ogu115-8A	CMS line	BC_13_	IARI Katrain	Absent
A63	Ogu310-8A	CMS line	BC_11_	IARI Katrain	Absent
A64	Ogu34-1-8A	CMS line	BC_12_	IARI Katrain	Absent
A65	Ogu34-8A	CMS line	BC_12_	IARI Katrain	Absent
A66	Ogu-HL-50A	CMS line	BC_11_	IARI Katrain	Absent
A67	Brahma	Hybrid	-	Sakata	Absent
A68	Ogu15A	CMS line	BC_9_	IARI Katrain	Absent
A69	Ogu17A	CMS line	BC_9_	IARI Katrain	Absent
A70	Ogu50A	CMS line	BC_9_	IARI Katrain	Absent
A71	Ogu60A	CMS line	BC_10_	IARI Katrain	Absent
A72	Casper	Hybrid	**–**	RijkZwaan	Absent
A73	KTCF-10A	Hybrid	**–**	Private seed	Absent
A74	Ponder	Hybrid	**–**	RijkZwaan	Absent
A75	SM	Hybrid	**–**	RijkZwaan	Absent
A76	Indam	Hybrid	**–**	IAHS	Absent

### PCR Amplification

Nineteen pairs of mitochondrial markers were used to detect nucleotide diversity among male sterile mitotypes of Indian cauliflowers (Supplementary Table 1) (Shu et al., 2016). One pair of primers (P1) was specific to *orf138* determining *Ogura* CMS, two pairs of primers were specific to *orf222* (P2 and P3), and one pair specific to *orf222-orf224* (P4), *orf224* (P5), *atp6-orf224* (P6), and *orf263* (P7). The six pairs of primers (P8–P13) were based on *orf138-*related genomic sequences, and other six pairs (P14–P19) were mitochondrial simple sequence repeat markers (mt-SSRs). The PCR amplifications were carried out in a reaction mixture of 25 μl consisted of 2 μl of genomic DNA template (50 ng), 1 μl of each forward and reverse primers, 12.50 μl of 2× PCR Green master mix (GoTaq DNA polymerase; Promega, United States), and 8.50 μl nuclease free water. The Eppendorf Mastercycler Nexus GSX1 was used for PCR amplification. Amplification was done following PCR cycling program: an initial denaturation of 95°C for 5 min, then 35 cycles of denaturation at 95°C for 30 s, annealing of primers at suitable temperature for 30 s and extension at 72°C for 1 min, and a final extension of 72°C for 7 min. The amplification by each polymorphic locus was repeated three times for the confirmation, and the PCR products were separated on 3% agarose gel electrophoresis in 1× TBE buffer (pH 8.0) at 100 mA voltage for 120 min. Ethidium bromide (EtBr) of 0.5 mg/ml was used for gel staining, and gel pictures were captured using digital gel documentation unit (BioSpectrum^®^ Imaging System^TM^, United Kingdom). The determination of fragment sizes was done using Promega^TM^ 100 bp DNA step ladder.

### Cluster Analysis Based on Mitochondrial Markers

For the cluster analysis based on mitochondrial markers, we studied the gel pictures and a binary matrix was used by converting the polymorphic loci data into 1 and 0. The amplified bands were scored visually as 1 for presence and 0 for absence. The qualitative differences in the band intensities were not taken into consideration for analysis. To categorize the CMS lines into distinct groups, the molecular data generated by combining the two types of analytical methods (mitochondrial DNA-specific markers and *B. napus*-based mt-SSRs) and subjected to cluster analysis *via* neighbor-joining (NJ) unweighted pair group method with arithmetic mean (UPGMA) using DARwin v6.0.017 (Perrier and Jacquemoud-Collet, 2006). The simple matching (SM) coefficient was computed to calculated genetic distance.

### Sequence Analysis

Initially, PCR amplification detected clear polymorphism for the primers P15 and P16 in terms of the length of amplified products. Based on the gel picture analysis, polymorphic fragments were selected for subsequent cloning and sequencing. Using polymorphic variants in the gel image, four representative polymorphic amplicons of both these groups of respective primers P15 and P16 were purified for sequencing. Two variants of each polymorphic fragment with different length were used for cloning and sequencing. In total, eight representative polymorphic amplicons (four from primer P15 and four from primer P16) were selected. Sequencing revealed that fragments amplified by primer P15 were of 251 bp in 46 CMS lines under study and 220 bp for the remaining CMS lines. The amplicons generated by primer P16 were of the size 420 bp in 42 CMS accessions under study and 471 bp in the remaining. The representative amplified PCR products were purified using Wizard^®^ SV Gel and PCR Clean-Up System (Promega). For each of the polymorphic amplified fragment, the representative purified PCR products were cloned into pGEM^®^-T Easy vector system (Promega Corporation, United States) following the supplier’s guidelines. The products were transformed into *Escherichia coli* (*DH5α*)-competent cells. The positive clones were confirmed by colony PCR and restriction digestion. The five positive clones for each of the selected polymorphic fragments were subjected to Sanger Dideoxy sequencing (Eurofins Genomics India Pvt., Ltd.). The percent sequence identity and similarity analysis was carried out with NCBI (National Center for Biotechnology Information) web-based BLAST service^1^ (Altschul et al., 1990) for the comparative analysis of obtained sequences with available relevant mitochondrial genome sequences in *Brassicaceae.* The Sanger sequences obtained were assembled and subjected to multiple sequence alignment with reference mitochondrial genomes to determine any variation at nucleotide level using SeqMan Pro tool of DNASTAR version 15.3^2^ (Lasergene Inc., Madison, WI, United States). The phylogenetic tree was constructed with MEGA X software (Kumar et al., 2018) based on different datasets of polymorphic primers. NCBI ORF finder^3^ was used to determine *ORF* numbers in the representative DNA sequence of polymorphic loci and to screen hypothetical protein translations. To verify predicted proteins, NCBI BLASTP or SMART BLAST (Altschul et al., 1990) was used. The amino acid multiple sequence alignment was performed using CLUSTAL Omega program (Sievers et al., 2011).

### Accession Numbers of Selected Polymorphic Amplicons

The sequences of selected polymorphic amplicons of Ogu307-33A, Ogu33-1A, Ogu16A, Ogu12A, Ogu12A-orf125, Ogu121-2A, Ogu-HL-3A, Ogu17A, and Ogu50A were submitted to the GenBank nucleotide sequence database of NCBI, and respective accession numbers were received after online publication as MN549523, MN549524, MN549525, MN549526, MN549527, MN549528, MN549529, MN549530, and MN549531, respectively. These accession numbers will be used hereafter.

### Impact of Cytonuclear Interactions on Floral-Nectar Phenotype

The field study was carried out at Baragram Experimental Farm of ICAR-IARI, Regional Station, Katrain, Himachal Pradesh, India, situated along the river Beas. The recommended package of practices, suggested for growing a healthy crop at the Baragram Experimental Farm, were followed for better agronomic and phenotypic expression of crop (Singh et al., 2019c). To determine the effect of cytonuclear interactions on floral reproductive traits, a comparative phenotypic analysis of a set of 60 CMS lines of snowball group of Indian cauliflower along with their respective male fertile counterparts (maintainer lines) was carried out based on important floral morpho-physiological traits viz. (i) petal color, (ii) shape of style, (iii) presence of floral nectaries, (iv) presence of viable pollen, (v) type of ovary, (vi) petal size: petal length (mm) and petal width (mm), (vii) ratio of petal length to petal width, (viii) sepal size: sepal length (mm) and sepal width (mm), (ix) ratio of sepal length to sepal width, (x) short filament length (mm), (xi) long filament length (mm), (xii) short stamen length (mm), (xiii) long stamen length (mm), (xiv) style length (mm), (xv) ratio of stamen to style, (xvi) ratio of long stamen to short stamen, and (xvii) nectar quantity (μl) (Singh and Srivastava, 2006; Chang et al., 2011; Gautam et al., 2011; Dey et al., 2017b; Kang et al., 2017). The petal length to petal width ratio was estimated to determine changes in petal size. The long stamen to style ratio was determined to estimate relative position of stigma to stamen. The long to short stamen ratio was estimated to determine the effect of cytoplasm on stamen length (Singh and Srivastava, 2006). The presence or absence of pollen grains was determined on the basis of visual observation, and pollen viability was tested by staining with 2% acetocarmine (Singh and Srivastava, 2006). All the CMS lines and their respective maintainers were evaluated for different floral traits in randomized block design (RBD) with three replications to quantify the modifications in flower reproductive features due to cytonuclear conflict. The data set was subjected to statistical analysis using paired *t*-test. Observations were recorded from 12 flowers per genotype (four flowers from three plants) in each replication. The CMS lines were compared with their respective maintainers for detection of floral abnormalities (e.g., pistillody, carpelloid stamen, petaloid stamens, splitted style, partially opened flowers, fused flowers, absence of nectaries, etc.). Floral nectar is an important trait for comprehending the plant-pollinator mutualisms. For the analysis of effect of nuclear-genomic conflict on nectar production, the quantification of nectar production was done from the CMS lines and their respective maintainers. The data was taken from 10 flowers of each genotype during early morning hours with graduated capillary of 10 μl. The average data was used for further statistical analysis.

### Statistical Analysis for Floral Reproductive Traits

All the CMS lines were clustered into different groups based on cluster analysis and dendrogram construction based on Euclidean distance was done with the PCA and NJ UPGMA method using DARwin software version 6.0.017 (Perrier and Jacquemoud-Collet, 2006). For testing the reliability of NJ dendrogram, a bootstrap value of 1,000 replicates was used. To compare the CMS lines with their respective male fertile counterparts, an average of CMS lines and their male fertile counterparts (maintainers) for different floral traits was analyzed using paired *t*-test by subjecting the data to “R” statistical software.

## Results

### Determining Cauliflower Cytoplasm Types and Genetic Divergence

**FIGURE 1:**
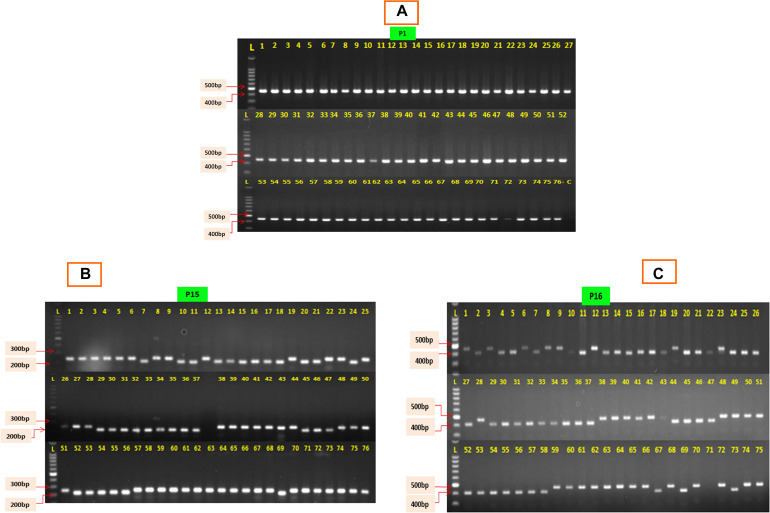
PCR amplification profile of 76 CMS lines of cauliflower. **(A)** The P1 depicts the PCR amplification with primer P1 specific to *orf-138*; L, ladder; C, control (Sel-27). **(B)** Amplification profile of 76 CMS lines exhibiting polymorphism with primer P15 (*BnTR4*). **(C)** Amplification profile of 76 CMS lines exhibiting polymorphism with primer P16 (*orf125*).

The 76 CMS accessions (63 indigenously developed CMS lines and 13 CMS based hybrids) of Indian cauliflowers (Table 1) were screened using 19 pairs of mitochondrial markers. These markers consist of 13 pairs of mitochondrial gene-specific primers: P1–P13 and six pairs of mt-SSRs, P14–P19, designed based on the *B. napus* mitochondrial genome (Supplementary Table 1) (Honma et al., 2011; Wang et al., 2012; Shu et al., 2016). Among the 13 pairs of mitochondrial gene-specific primers, seven pairs specific to *orf138* were amplified. They did not show any amplification in the control inbred line, Sel-27 devoid of *Ogura* cytoplasm (Figure 1A). However, none of the primers specific to *pol*, *nap*, and *tour* cytoplasms exhibited any amplification in the CMS lines. The six pairs of mitochondrial SSRs were amplified across the 76 Indian cauliflower CMS lines. The amplification pattern of the gene-based primers indicated that the cytoplasm type of all the CMS lines in Indian cauliflower was derived from *Ogura* cytoplasm; however, there was a variation at nucleotide level among the large genetic stock of Indian cauliflower. These results indicated presence of only *Ogura* cytoplasm in all the cauliflower lines and F_1_ hybrids under study, and none of the CMS lines and F_1_ hybrids were based on other types of cytoplasmic systems. Thus, these set of primers can be utilized effectively across the *B. oleracea* CMS germplasm to distinguish the diverse CMS systems harboring different cytoplasm types. However, different cytoplasm type-based CMS system is widely utilized across the *Brassica* crops and different cytoplasms are associated with different phenotypes. Thus, these mitochondrial markers would be helpful to distinguish CMS systems for their effective use in hybrid breeding program. Two pairs of mt-SSRs, P15 (*BnTR4*) and P16 (*orf125*) exhibited striking polymorphism among the CMS lines (Figures 1B,C). The amplicon size for the primers P15 and P16 varied between 200 and 280 bp and 410 and 470 bp, respectively (Figures 1B,C).

The principal component analysis (PCA) and NJ cluster analysis based on mitochondrial markers were used to estimate the diversity in origin of male sterile cytoplasm in cauliflower lines. The cluster analysis of the cauliflower CMS lines categorized them into three major distinct groups each with two subgroups and further subclusters in each subgroup (Supplementary Figures 1a,b). Broadly, six distinct subgroups were determined based on the origin of their cytoplasm source (Supplementary Figure 1b). The six CMS lines (A38 to A43) including four CMS-based hybrids CFH1522, Kimaya, Pahuja, and Snowpearl remained in one single group; the other eight CMS lines (A44 to A51) belonged to a different group. This group comprised four CMS-based hybrid from private seed companies; it might be that same original source of *Ogura* cytoplasm was used to develop female parent of these hybrids. Ten CMS lines (A52 to A61) belonged to the third group and other 15 CMS lines (A62 to A76) including six CMS-based hybrids (SM, Indam, KTCF-10A, Casper, Ponder, Brahma) formed a distinct fourth group. The nine CMS accessions (A29 to A37) represented in fifth group and the rest of the 28 CMS lines (A1 to A28) including three CMS-based hybrids of Acsen HyVeg private limited (HVCF-29, HVCF-18, and HVCF-16) formed the largest group (Supplementary Figure 1b). These results indicated that all the CMS lines are based on *Ogura* cytoplasm, but their source of origin might be different. Besides, cytonuclear genomic incompatibilities played a significant role in genetic variation during the course of speciation of these lines.

### Sequence Analysis

**FIGURE 2:**
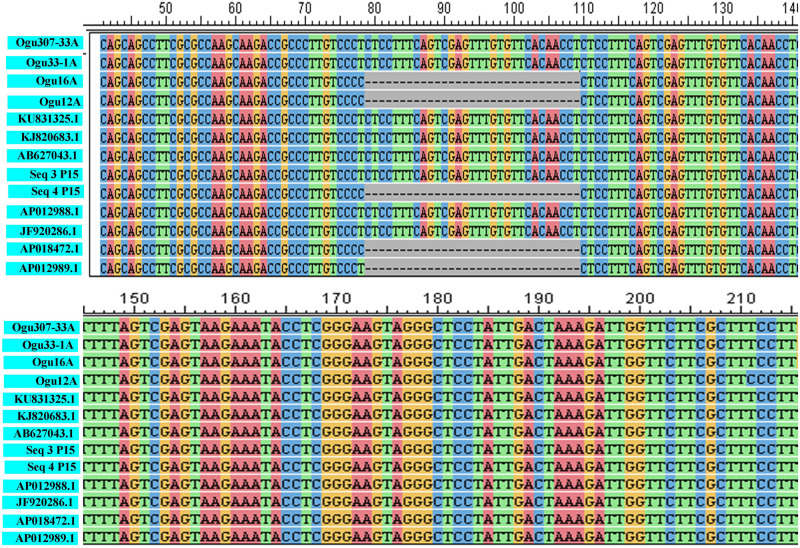
Sequence analysis and alignment of polymorphic amplicons generated by primer P15.Ogu307-33A (MN549523), Ogu33-1A (MN549524), Ogu16A (MN549525), and Ogu12A (MN549526) are selected polymorphic amplicons of CMS lines. Numbers in parenthesis are accession numbers obtained for the respective fragment sequence submitted to GenBank. KU831325.1 (*Brassica oleracea* var. *capitata*), KJ820683.1 (*Brassica oleracea* var. *botrytis*), AB627043.1 (*Brassica oleracea*), AP012988.1 (*Brassica oleracea*), JF920286.1 (*Brassica oleracea*), AP018472.1 (*Raphanus sativus*), and AP012989.1 (*Brassica nigra*) are related reference mitochondrial genome sequences of *Brassicaceae* crops available in NCBI gene bank database. Seq3 P15 and Seq4 P15 are reference sequences of broccoli from Shu et al. (2016).

The fragments amplified by primer P15 were of 251 bp for 46 CMS lines under study and 220 bp for the remaining CMS lines. The representative polymorphic amplicons of both these groups depicting 251 and 220 bp sizes were selected randomly based on the size of the amplicons. They were purified and cloned in pGEM-T vector and sequenced. The obtained sequences were subjected to multiple sequence alignment with each other and reference mitochondrial genome sequences of *Brassicaceae* crops exhibiting high degree of sequence similarity (Table 2). The accession numbers for the sequenced fragments were obtained from GenBank NCBI as described in the Section “Materials and Methods.” The sequence analysis revealed that MN549523 shared highly conserved region with MN549524 and high degree of similarity with other reference genomic regions of mitochondrial genome sequences of KU831325.1, KJ820683.1, AB627043.1, Sequence 3 P15 from Shu et al. (2016), AP012988.1, and JF920286.1 (Figure 2 and Table 2). Whereas, the MN549525 exhibited high degree of similarity with MN549526 and reference mitogenome sequences used in sequence alignment analysis such as Sequence 4 P15 from Shu et al. (2016), AP018472.1, and AP012989.1 (Figure 2). The sequence alignment revealed a single nucleotide polymorphism (SNP) at position 78 (C/T) and in addition, the deletion of 31 nucleotide between positions 78 and 110 for MN549525 and MN549526 (Figure 2).

**TABLE 2:** Sequence similarity percentage of the selected polymorphic amplicons of cauliflower CMS accessions generated by mt-SSR (*BnTR4*) with corresponding mitogenome sequences.

**Accession No.**	**Species**	**Description**	**Sequence identity (Ogu307-33A)**	**Sequence identity (Ogu33-1A)**	**Sequence identity (Ogu16A)**	**Sequence identity (Ogu12A)**
KU831325.1	*Brassica oleracea* var. *capitata*	Mitochondrion, complete genome	97.12%	97.12%	96.60%	95.24%
KJ820683.1	*Brassica oleracea* var. *botrytis*	Mitochondrion, complete genome	97.12%	97.12%	96.60%	95.24%
AB627043.1	*Brassica oleracea*	Mitochondrial DNA, minisatellite: BnTR4	99.47%	98.95%	99.12%	99.10%
AP012988.1	*Brassica oleracea*	Mitochondrial DNA, complete sequence, cultivar: Fujiwase	97.12%	97.12%	96.60%	95.24%
JF920286.1	*Brassica oleracea*	Mitochondrial DNA, complete genome	97.12%	97.12%	96.60%	95.24%
AP018472.1	*Raphanus sativus*	Mitochondrial DNA, complete genome, cultivar: Kosena	95.86%	95.77%	96.79%	95.87%
AP012989.1	*Brassica nigra*	Mitochondrial DNA, complete sequence	96.55%	95.86%	96.33%	95.41%

**FIGURE 3:**
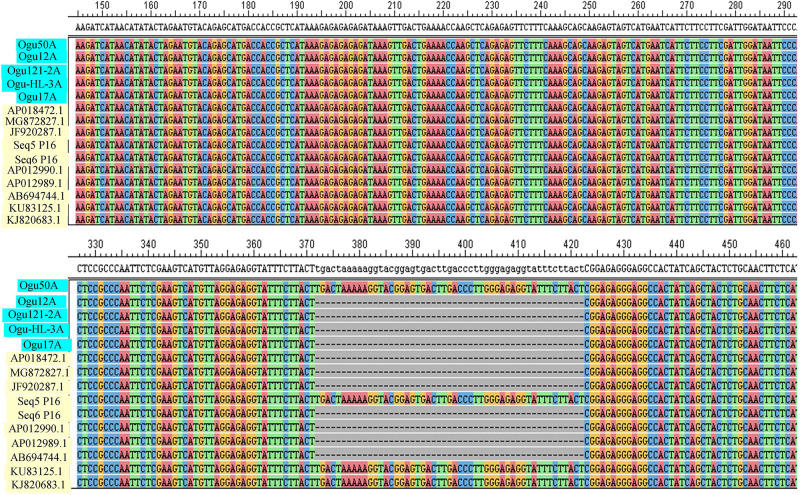
Sequence analysis and alignment of polymorphic amplicons generated by primer P16. Ogu50A (MN549531), Ogu12A-orf125 (MN549527), Ogu17A (MN549530), OguHL-3A (MN549529), and Ogu121-2A (MN549528) are selected polymorphic amplicons of cauliflower CMS mitotypes. Numbers in parenthesis are accession numbers obtained for the respective fragment sequence submitted to GenBank.KU831325.1 (*Brassica oleracea* var. *capitata*), KJ820683.1 (*Brassica oleracea* var. *botrytis*), AB694744.1 (*Raphanus sativus*), AP012990.1 (*Raphanus sativus*), JF920287.1 (*Brassica carinata*), AP018472.1 (*Raphanus sativus*), AP012989.1 (*Brassica nigra*), and MG872827.1 (*Brassica juncea*) are related reference mitochondrial genome sequences of *Brassicaceae* crops available in NCBI gene bank database. Seq5 and Seq6 P16 are reference sequences of broccoli from Shu et al. (2016).

The amplicons generated by primer P16 were of size 420 bp in 42 CMS accessions under study and 471 bp in the remaining. The obtained sequences were subjected to multiple sequence alignment with other mitogenome sequences of *Brassicaceae* crops available in NCBI database (Table 3). The sequence similarity analysis revealed that amplicons of MN549527 to MN549530 shares a high degree of sequence similarity with each other and CMS-related proteins in other *Brassicaceae* accessions AP018472.1, MG872827.1, JF920287.1, Seq6 P16 from Shu et al. (2016), AP012990.1, AP012989.1, and AB694744.1 (Figure 3). Likewise, MN549531 exhibited highly conserved region with Seq5 P16 from Shu et al. (2016), KU831325.1, and KJ820683.1 (Figure 3). The sequence alignment analysis revealed a deletion of size 51 bp between positions 371 and 423 in the exonic regions of *ORFs* for MN549527 to MN549530. While normal sequence without deletion was observed for MN549531.

**TABLE 3:** Sequence similarity percentage of the selected polymorphic amplicons of cauliflower CMS accessions generated by mt-SSR (*orf125*) with corresponding mitogenome sequences.

**Accession No.**	**Species**	**Description**	**Sequence identity (Ogu50A)**	**Sequence identity (Ogu12A)**	**Sequence identity (Ogu121-2A)**	**Sequence identity (Ogu17A)**
KU831325.1	*Brassica oleracea* var. *capitata*	Mitochondrion, complete genome	99.34%	99.06%	99.06%	99.36%
KJ820683.1	*Brassica oleracea* var. *botrytis*	Mitochondrion, complete genome	99.34%	99.06%	99.06%	99.36%
AB694744.1	*Raphanus sativus*	Mitochondrial DNA, complete genome, cultivar: MS-gensuke	99.08%	98.82%	98.58%	96.88%
AP012990.1	*Raphanus sativus*	Mitochondrial DNA, complete sequence, cultivar: Black radish	99.39%	98.58%	98.35%	96.63%
JF920287.1	*Brassica carinata*	Mitochondrial DNA, complete genome	99.39%	98.58%	98.35%	96.63%
AP018472.1	*Raphanus sativus*	Mitochondrial DNA, complete genome, cultivar: Kosena	99.08%	98.82%	98.58%	96.88%
AP012989.1	*Brassica nigra*	Mitochondrial DNA, complete sequence	99.39%	98.58%	98.35%	96.63%
MG872827.1	*Brassica juncea*	Isolate 93 mitochondrion, complete genome	99.39%	98.58%	98.35%	96.63%

### Cytoplasmic Genetic Variations and Floral Malformations

**FIGURE 4:**
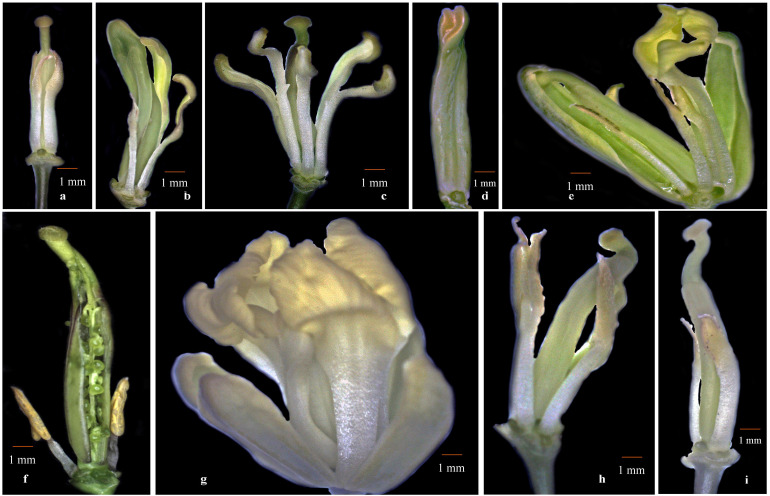
Floral deformities associated with cyto-nuclear conflict. **(a)** Adherence of functional stamens with style, **(b)** petaloidy condition of stamens, **(c)** partial petaloidy of functional stamens, **(d)** splitted style along with adherence of stamens, **(e)** stigma hidden inside the petals and petaloid stamens, **(f)** splitted style with exposed ovules, **(g)** unopened flower, and **(h**,**i)** stamens adherence with style and bent stigma.

We sought to investigate whether cytoplasmic genetic diversity associated with SNPs or InDels at loci *BnTR4* and *orf125* (Figures 2, 3) has specifically impacted floral phenotypes of CMS lines. It was evident that the CMS lines exhibiting SNPs and deletions of 31 or 51 nucleotides in the *ORFs* exonic region was associated with varying degrees of floral abnormalities across the whole set of the CMS lines (Figure 4 and Table 4). The CMS lines with the absence of these deletions of nucleotides identified by polymorphic markers had normal flower structure with no deformities. More than 80% of the plants among CMS lines with deletions had one or another floral malformations indicating deformed flower structure. However, extent and type of deformities varied among the genotypes. The major deformities recorded in the CMS lines were (i) adherence of functional stamens with style, (ii) homeotic-like floral transformation petaloidy condition of stamens, (iii) partial petaloidy of functional stamens, (iv) splitted style along with adherence of stamens, (v) stigma hidden inside the petals, (vi) splitted style along with exposed ovules, (vii) unopened flower, (viii) stamens adherence with style and crooked stigma, (ix) partially opened flowers, (x) absence of nonfunctional stamens, (xi) fused flower, (xii) curved functional stamens with crooked stigma, and (xiii) absence of nectaries. Most of the CMS lines with none of the above floral deformities had normal female fertility and seed set (Figure 4 and Table 4).

**TABLE 4:** Impact of cytoplasmic genetic variations based on InDels on floral phenotypes in varying nuclear backgrounds.

**Code**	**CMS accessions**	**Type of nucleotide variation (SNPs/deletions)**		**Flower phenotype of cytolines (CMS)**	
		**(*BnTR4*)**	**(*Orf125*)**	**Normal**	**Varying abnormalities**
A1	Ogu122-5A	–	–	NCMS	
A2	Ogu115-33A	–	1 (51)		A
A3	Ogu118-6A	–	–	NCMS	
A4	Ogu307-33A	1 (T/C)	1 (51)		A, B, F, G
A5	Ogu33-1A	1 (T/C)	1 (51)		A, B, G
A7	Ogu309-2A	1 (31)	1 (51)		A, F, G
A8	OguKt-2-6A	–	–	NCMS	
A9	Ogu13-85-6A	–	–	NCMS	
A10	Ogu16A	1 (C/T), 1 (31)	1 (51)		B, F, J
A11	Ogu3A	1 (31)	1 (51)		B, J
A12	Ogu2-6A	–	–	NCMS	
A13	Ogu2A	1 (31)	1 (51)		F, I, J
A14	Ogu14A	1 (31)	1 (51)		J
A15	Ogu122-1A	1 (31)	1 (51)		J
A16	OguKt-9-2A	1 (31)	1 (51)		J, K
A17	Ogu121-1A	1 (31)	1 (51)		J
A18	Ogu126-1A	1 (31)	1 (51)		J
A19	Ogu134-8A	–	–	NCMS	
A20	Ogu12A	1 (C/T), 1 (31)	1 (51)		F, I, J
A21	Ogu119-1A	–	1 (51)		A
A22	Ogu34A	–	1 (51)		A
A23	Ogu178-8A	–	–	NCMS	
A24	Ogu118-2A	1 (31)	1 (51)		G, J
A25	Ogu33A	–	1 (51)		A
A29	OguKt-2-1A	1 (31)	1 (51)		J, K
A30	Ogu309-1A	1 (31)	1 (51)		I, J
A31	Ogu-HL-1A	1 (31)	1 (51)		L
A32	Ogu307-1A	1 (31)	1 (51)		A
A33	Ogu Kt-8-2A	1 (31)	1 (51)		J, K
A34	Ogu119-2A	1 (31)	1 (51)		A
A35	Ogu121-2A	1 (31)	1 (51)		I, J, K
A36	Ogu-HL-3A	1 (31)	1 (51)		A, C
A37	Ogu13-85-3A	1 (31)	1 (51)		B, I
A38	Ogu119-6A	–	**–**	NCMS	
A43	Ogu13A	–	**–**	NCMS	
A44	Ogu34-1A	–	1 (51)		A
A45	Ogu1-2A	1 (31)	1 (51)		I
A46	Ogu13-85-2A	1 (31)	1 (51)		B, J
A47	Ogu118-3A	1 (31)	1 (51)		B, J
A48	Ogu307-6A	–	**–**	NCMS	
A49	Ogu-HL-6A	–	**–**	NCMS	
A50	Ogu308-6A	–	**–**	NCMS	
A51	Ogu13-01-5A	–	**–**	NCMS	L
A52	Ogu13-85-4A	1 (31)	1 (51)		M
A53	Ogu118-4A	1 (31)	1 (51)		B, K
A54	Ogu76-4A	1 (31)	1 (51)		A, K
A55	Ogu33-4A	1 (31)	1 (51)		A, K
A56	Ogu77-4A	1 (31)	1 (51)		B, C, E
A57	Ogu13-85-33A	–	1 (51)		A
A58	Ogu76-33A	–	1 (51)		A
A59	Ogu122-8A	–	–	NCMS	
A60	Ogu1-8A	–	–	NCMS	
A61	Ogu309-8A	–	–	NCMS	
A62	Ogu115-8A	–	–	NCMS	
A63	Ogu310-8A	–	–	NCMS	
A64	Ogu34-1-8A	–	–	NCMS	
A65	Ogu34-8A	–	–	NCMS	
A66	Ogu-HL-50A	–	–	NCMS	
A68	Ogu15A	–	–	NCMS	
A69	Ogu17A	1 (31)	1 (51)		L
A70	Ogu50A	–	–	NCMS	
A71	Ogu60A	–	–	NCMS	

*(A) Adherence of functional stamens with style; (B) homeotic-like floral transformation: petaloidy condition of stamens; (C) partial petaloidy of functional stamens; (D) splitted style along with adherence of stamens; (E) stigma hidden inside the petals; (F) splitted style along with exposed ovules; (G) unopened flower; (H) stamens adherence with style and crooked stigma; (I) partially opened flowers; (J) absence of non-functional stamens; (K) fused flower; (L) very curved functional stamens with crooked stigma; (M) absence of nectaries; (NCMS) cytoplasmic male sterile sources with normal flower phenotype and devoid of other abnormalities as mentioned in A–M. Although, the conventional changes in flower morphology owing to cyto-nuclear interaction are evident; (51) The number in parenthesis under the column deletions denote the number of base pair deletions, 1 denotes the numbers of SNP (means one SNP).*

### Analysis of *ORFs* and Similarity of Genes, Phylogenetic Relationships

**FIGURE 5:**
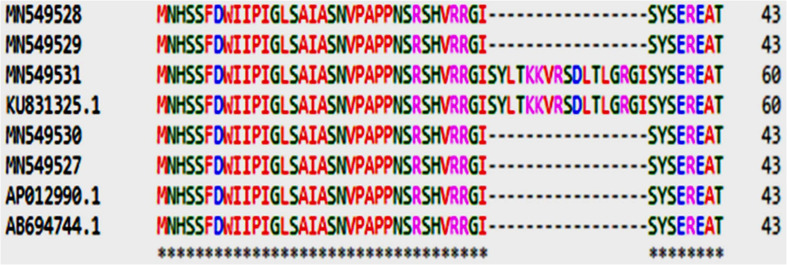
Amino acid sequence analysis of *ORFs* of P16 polymorphic amplicons. MN549531, MN549527, MN549530, MN549529, and MN549528 are selected protein sequences of *ORFs* of cauliflower CMS mitotypes. KU831325.1 (*Brassica oleracea* var. *capitata*), AB694744.1 (*Raphanus sativus*), and AP012990.1 (*Raphanus sativus*) are reference amino acid sequences of *Brassicaceae* obtained by NCBI ORF finder.

The ORF analysis revealed nucleotide deletion in the *orf125* coding region of the male sterile cytoplasm. The protein sequence analysis of polymorphic amplicons and reference mitotypes showed deleted nucleotide-encoded 17 amino acids (Figure 5). Therefore, polymorphism of P15 (*BnTR4*) and P16 (*orf125*) could be ascribed to SNPs or fragment insertion/deletion near the mt-SSR loci. The amino acid sequence analysis in the region of *ORF* depicted the polymorphic region amplified by P16 mt-SSR which is located in the coding region of *orf125* protein of *B. oleracea* (wild cabbage), *B. juncea, B. rapa* ssp. *oleifera, Eruca vesicaria* subsp. s*ativa*, *B. oleracea* var. *capitata*, and *B. oleracea* var. *botrytis* (Supplementary Table 2) besides its location in *B. carinata* exonic region of *orf108c.* The protein sequence analysis revealed deletion of amino acids related to array of floral deformities with high degree of similarity with *orf108c* in *B. carinata* (Supplementary Table 2). The protein sequences devoid of deletion exhibited high similarity with *orf125* in *B. oleracea.*

**FIGURE 6:**
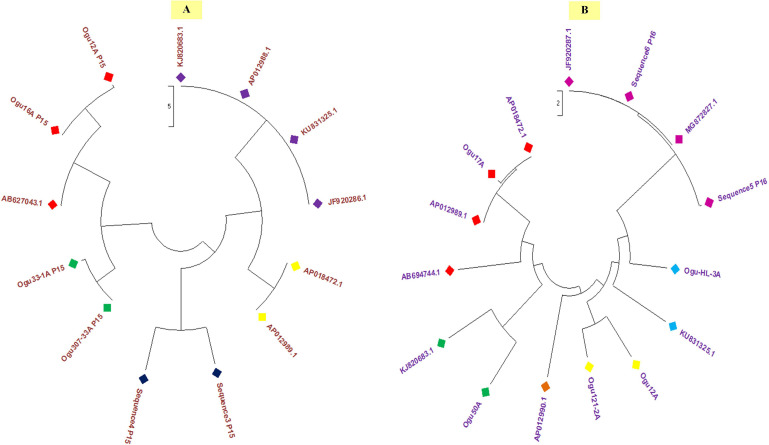
Phylogenetic analysis. **(A)** Phylogenetic tree based on sequence analysis of selected polymorphic fragments generated with primer P15 and related reference mitogenome sequences from NCBI gene bank database. **(B)** Phylogenetic tree based on sequence analysis of selected polymorphic amplicons generated with primer P16 and related reference mitogenome sequences from NCBI gene bank database.

Polymorphism exhibited with P15 mt-SSR and mt-DNA sequences of other nine *Brassicaceae* mitotypes were broadly grouped into two major groups (Figure 6A). The phylogenetic analysis revealed a close affinity of cauliflower CMS lines, Ogu16A with Ogu12A and Ogu307-33A with Ogu33-1A. These cytoplasmic sources were clustered in one major group with one diploid accession of *B. oleracea.* This clustering in one group concurs with *rapa*/*oleracea* lineage propounded by Warwick and Black (1991) based on chloroplast genome. Another group represented diploid *B. oleracea* var. *botrytis*, *B. oleracea* var. *capitata*, *B. oleracea* var. *italica*, *Raphanus sativus* var. *kosena*, and *B. nigra.* This grouping also corresponded to *rapa*/*oleracea* lineage as proposed by Warwick and Black (1991) except one species of *nigra* lineage. Another group also confined to *rapa*/*oleracea* lineage with the exception of one species of *nigra* lineage. Similarly, the polymorphic amplicons based on P16 mitochondrial marker and corresponding mt-DNA sequences of 10 *Brassicaceae* mitotypes were clustered in two major groups (Figure 6B). The selected CMS sources, Ogu50A and Ogu17A were distantly placed from other CMS sources. OguHL-3A, Ogu12A, and Ogu121-2A exhibited a close affinity with each other and *B. oleracea* var. *capitata* based on phylogenetic analysis. The CMS line Ogu50A depicted high affinity with KJ820683.1 (*B. oleracea* var. *botrytis* L.) and Ogu17A showed high affinity with mitogenome of *B. nigra* and *R. sativus* var. *kosena*, and clustered in one major group along with *Raphanus sativus* cv. Ms-gensuke, Ogu50A, and *B. oleracea* var. *botrytis* (KJ820683.1). The black radish distantly placed itself from other members of its group.

### Impact of Cytonuclear Interactions on Floral-Nectar Phenotypes

#### Floral Qualitative Traits

In the present investigation, we sought to analyze whether cytonuclear interactions specifically affected floral qualitative traits in the CMS lines of Indian cauliflowers. Cauliflower CMS lines along with their respective maintainers were characterized for five traits namely style shape, petal color, presence of floral nectaries, presence of viable pollen, and type of ovary (Supplementary Table 3 and Supplementary Figures 2, 3). All the CMS lines had normal ovary and varied nectaries. None of the CMS line showed the presence of viable pollen grains and few lines were devoid of anther and pollen grains. The CMS accessions had straight to slightly curved or fully curved stigma. Yellow-colored petals were predominant; however, white petals were recorded in Ogu33A, Ogu134-8A, Ogu13A, Ogu118-6A, OguKt-2-6A, and Ogu118-2A.

### Cytonuclear Interactions Influencing Floral Reproductive Phenotypes

Comparative analysis for floral reproductive whorls and floral phenotypes of cauliflower CMS lines and their respective male fertile counterparts was conducted to determine the effects of mitonuclear coadaptation and disruption. The *per se* performance of several CMS lines and their respective maintainers recorded significant variation for different floral phenotypes (Supplementary Table 4 and Supplementary Figure 3). Petal length varied from 12.91 mm (Ogu76-4A) to 17.92 mm (Ogu2-6A). The longest petal was recorded in the CMS line Ogu2-6A followed by Ogu126-1A and Ogu310-8A. The petal width ranged from 4.18 mm (Ogu13-85-2A) to 7.83 mm (Ogu2-6A). The CMS line, Ogu2-6A followed by Ogu178-8A and Ogu33A-1301 had the widest petals. The ratio of petal length to petal width varied from 1.88 (Ogu33A-1301) to 3.82 (Ogu13-85-2A), and ratio was >2 except for Ogu-13-01-5A, Ogu307-1A, and Ogu-13-01-33A. The considerable differences were also observed for sepal size, and the sepal length and width varied from 6.23 mm (Ogu15A) to 10.28 mm (Ogu2-6A) and 2.21 mm (Ogu13-85-2A) to 3.53 mm (Ogu115-8A), respectively. The ratio of sepal length to sepal width ranged from 2.19 (Ogu15A) to 3.88 (OguKt-2-1A). Like petal size, sepal size too was significantly reduced in the male sterile lines. The length of short (nonfunctional) and long (functional) filament ranged from 2.30 mm (Ogu16A) to 6.38 mm (Ogu310-8A) and 3.40 mm (33A-1301) to 7.52 mm (Ogu310-8A), respectively. The introgression of sterile cytoplasm resulted in the elimination of nonfunctional filaments in nine CMS lines. Marked differences were also observed for stamen length, as the short stamen (nonfunctional) length varied from 3.41 mm (Ogu16A) to 8.13 mm (Ogu310-8A) and long stamen (functional) length from 4.79 mm (33A-1301) to 9.48 mm (Ogu2-6A). The ratio of functional (long stamen) to nonfunctional anthers (short stamen) was determined to detect any changes in CMS types, and it varied from 1.03 (OguKt-2-1A) to 2.07 (Ogu77-4A) and from 1.03 to 1.26 in maintainer lines. The nine CMS lines *viz*. Ogu121-1A, Ogu122-1A, Ogu126-1A, OguKt-9-2A, OguKt-8-2A, Ogu118-3A, Ogu2A, Ogu3A, and Ogu118-2A were completely devoid of nonfunctional stamens. The significant differences were also observed in all the CMS lines for style length and varied from 5.32 mm (Ogu14A) to 9.44 mm (Ogu16A). However, in the male fertile counterparts, the style length varied from 6.33 to 10.86 mm. These results clearly indicated the effect of cytonuclear genomic incompatibilities on reproductive whorls. Reduction in style length, stamen length, and even absence of short stamens was also recorded in the CMS lines in different nuclear backgrounds. Generally, the position of style was higher than the stamens in majority of the CMS lines except Ogu-Kt-2-1A, Ogu122-5A, Ogu118-4A, Ogu33-1A, Ogu33A, Ogu77-4A, OguKt-9-2A, Ogu3A, and Ogu14A (Supplementary Table 4). The cytonuclear conflict invariably reduced the stamen length in all the Indian cauliflower lines. The relative position of stamens and stigma was determined by estimating the ratio between functional stamens and style length. The ratio varied from 0.62 (33A-1301) to 1.30 (Ogu14A) in CMS lines and 0.98 to 1.46 in male fertile counterparts.

### Impact of Cytonuclear Interactions on Nectar Production

Comparative nectar quantity analysis in the CMS lines and their respective male fertile maintainers was conducted to determine the impact of cytonuclear interactions for this trait. Significant variation was observed in the CMS lines for nectar quantity (Table 5). The nectar quantity varied from 0.28 μl (Ogu15A) to 5.69 μl (Ogu1-8A) in the CMS lines, while in the male fertile counterpart, the nectar quantity varied from 0.60 to 13.94 μl. The highest quantity of nectar was found in the CMS line, Ogu1-8A followed by Ogu308-6A and Ogu2-6A. The results revealed a significant reduction in the nectar quantity of CMS lines as compared with their respective male fertile counterparts, indicating that cytonuclear interactions caused inefficient nectar production (Table 5).

**TABLE 5:** Impact of cytonuclear interactions on nectar production of cytolines in varying nuclear backgrounds.

**A and B lines**	**Nectar quantity (μl)**	**% Nectar reduction in cytolines**	**CMS vs. maintainers**	**Nectar quantity (μl)**	**% Nectar reduction in cytolines**	**A and B lines**	**Nectar quantity (μl)**	**% Nectar reduction in cytolines**
Ogu33-1A	0.44**	73.17	Ogu1A	1.53**	85.57	Ogu2-6A	3.66**	65.50
Kt-33B1	1.64		Kt-1B	10.61		Kt-1B	10.61	
Ogu33A	0.38**	78.28	Ogu12A	2.24**	83.93	Ogu115-33A	2.02*	30.34
Kt-33B	1.75		Kt-12B	13.94		Kt-15B	2.90	
Ogu34-1A	0.60**	75.6	Ogu16A	0.44*	84.82	Ogu307-33A	0.87*	73.47
Kt-34B (WF)	2.46		Kt-16B	2.90		Kt-307-33B	3.28	
Ogu34A	0.65*	67.82	OguKt-2-1A	0.49*	84.82	Ogu1-8A	5.69**	46.37
Kt-34B (YF)	2.02		Kt-2B	3.23		Kt-1B	10.61	
Ogu15A	0.28*	86.13	Ogu121-1A	1.42	15.97	Ogu13-85-3A	0.55*	49.54
Kt-15B	2.02		Kt-121B	1.69		Kt-1385B	1.09	
Ogu17A	0.66**	76.76	Ogu121-2A	0.33*	76.76	Ogu2A	2.24*	30.65
Kt-17B	2.84		Kt-121B	1.42		Kt-2B	3.23	
Ogu119-1A	1.37*	32.17	Ogu1-2A	0.38*	88.23	Ogu3A	0.55*	81.35
RSK-119B	2.02		Kt-2B	3.23		Kt-3B	2.95	
Ogu307-1A	1.48**	54.87	Ogu-77-6A	0.55*	56.34	Ogu13A	1.59**	53.09
Kt-307B	3.28		Kt-77	1.26		Kt-13B	3.39	
Ogu309-1A	0.60**	64.49	Ogu-HL-6A	0.40**	90.74	Ogu14A	0.55**	85.21
Kt-309B	1.69		HL	4.32		Kt-14B	3.72	
Ogu-HL-1A	0.49**	88.65	Ogu122-5A	1.91**	74.66	Ogu119-2A	0.55*	72.77
HL	4.32		Kt-22B	7.54		RSK-119B	2.02	
Ogu309-2A	0.93*	44.97	Ogu122-8A	0.98**	87.00	Ogu13-85-2A	0.55	49.54
Kt-309B	1.69		Kt-22B	7.54		Kt-1385B	1.09	
Ogu1301-5A	3.61*	24.15	Ogu309-8A	2.35	10.30	Ogu118-2A	0.71*	73.99
Kt-1301B	4.76		Kt-309-8B	2.62		Kt-18B	2.73	
Ogu-HL-3A	0.55**	87.26	Ogu118-4A	0.44*	83.88	Ogu119-6A	0.38*	81.18
HL	4.32		Kt-18B	2.73		RSK-119B	2.02	
Ogu76-4A	1.09*	70.21	Kn81.1301	0.54**	88.65	Ogu13-85-6A	0.98	10.09
DC-76	3.66		Kt-1301B	4.76		Kt-1385B	1.09	
Ogu76-33A	1.04*	71.58	Ogu13-85-33A	0.44*	59.63	Ogu118-6A	0.87*	68.13
DC-76	3.66		Kt-1385B	1.09		Kt-18B	2.73	
Ogu77-4A	0.66*	47.61	Ogu122-1A	0.49**	93.50	OguKt-2-6A	0.86*	68.13
Kt-77B	1.26		Kt-22B	7.54		Kt-2B	2.73	
Ogu310-8A	0.60	26.82	Ogu126-1A	2.95**	78.76	Ogu-13-01-33A	1.37*	71.21
Kt-310B	0.82		Kt-126B	13.89		Kt-1301B	4.76	
Ogu178-8A	1.86*	35.86	OguKt-9-2A	1.04**	83.75	Ogu115-8A	1.05*	48.01
Kt-178B	2.90		Kt-9B	6.40		Kt-15B	2.02	
Ogu-HL-50A	0.66**	84.72	OguKt-8-2A	1.04**	91.61			
HL	4.32		Kt-8B	12.41				
Ogu13-85-4A	0.66*	39.44	Ogu118-3A	0.87*	68.13			
Kt-1385B	1.09		Kt-18B	2.73				
Ogu34-1-8A	0.60*	75.60	Ogu308-6A	5.30*	17.18			
Kt-34B (WF)	2.46		Kt-308B	6.40				

*Significance at 5% level of probability; **Significance at 1%.

### Cluster Analysis

**FIGURE 7:**
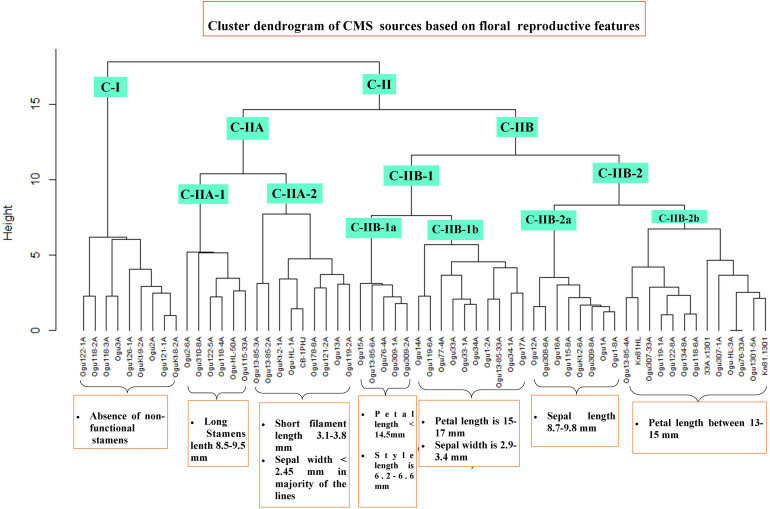
Cluster dendrogram of cauliflower cytoplasmic sources based on floral traits. Cluster C-I harbor all the CMS lines without nonfunctional stamens. Cluster C-II comprises different subclusters with varying degree of floral phenotypic variability and abnormalities.

The CMS lines were grouped into different clusters based on floral reproductive whorls and phenotypic traits, and two major clusters (CI and CII) were formed with two subclusters in cluster II (Figure 7). The subclusters (C-IIA and C-IIB) in cluster II were further grouped into subclusters C-IIA-1, C-IIA-2, C-IIB-1, and C-IIB-2. Majority of CMS lines grouped into clusters II and I had only nine CMS lines. All the CMS accessions with the absence of nonfunctional stamens remained in cluster I. The six CMS lines with functional stamen lengths of 8.5 to 9.48 mm remained in the subcluster C-IIA-1. The nine CMS accessions grouped into subcluster C-IIA-2 with nonfunctional filament length of 3.1–3.8 mm, and majority of the CMS lines in this cluster had sepal width of <2.45 mm. The subcluster C-IIB-1a contained five CMS lines with petal length of <14.5 mm, style length of 6.28–6.6 mm, and functional stamen length of 6.49–6.61 mm. The subcluster C-IIB-1b had 10 CMS lines, and majority of them (60%) have petal length of 15–17 mm and sepal width of 2.9–3.4 mm. The subcluster C-IIB-2a comprised eight CMS lines, and majority of them had sepal length of >8.7 mm (i.e., 8.7–9.8 mm). The remaining 13 CMS lines were clustered into subcluster C-IIB-2b with petal length of >13 to <15 mm in majority of the lines.

## Discussion

The CMS phenotype is a common feature in higher plants controlled by mt-DNA and widely used to facilitate hybrid development in *Brassica* vegetables (Shu et al., 2016; Singh et al., 2018a, 2019a; Dey et al., 2019). The propensity to frequent recombination, mt-DNA rearrangements, and encoding of numerous genes makes plant mitogenome more complex relative to their metazoan counterpart. In this context, the role of mitochondrial markers in quick identification and differentiation of different CMS types has been reported earlier in other members of *Brassicaceae* (Wang et al., 2012; Shu et al., 2015, 2016). We are reporting the usefulness of mitochondria-specific markers in selecting CMS lines with normal flower structure and extent of mitogenome diversity for the first time in Indian cauliflowers. In this study, mt-DNA-specific primers associated with different CMS genes, *orf138*, *orf222*, *orf224*, *orf222-224*, *orf263*, *atp6-orf224*, and mt-SSRs were utilized. The results revealed that all the CMS lines of Indian cauliflower possessed *orf138*-related fragment specific to *Ogura* CMS establishing the wide use of *Ogura* cytoplasm in the development of CMS system (Chen et al., 2009; Han et al., 2018; Singh et al., 2019a). Therefore, it is urgently needed to diversify the source of the CMS in Indian cauliflowers to avoid any imminent threat of epidemic which may collapse the entire hybrid seed industry of *Brassica oleracea* vegetables (Dey et al., 2013). The polymorphic mt-SSR loci indicated the presence of cytoplasmic genetic variation among *Ogura* CMS-based male sterile genetic stock of Indian cauliflowers. The PCA and NJ cluster analysis based on combined analysis of mt-DNA-specific and mt-SSR markers classified the 76 cytolines into six different groups (Supplementary Figure 1B).

The polymorphic amplicons generated with primer P15 were of size between 220 and 280 bp, while for P16 of 410–470 bp size. The lengths of polymorphic products of A13 to A17, A24 to A27, A29 to A37, A69, A73, A2, A4–A5, and A52–A58 were identical as reported in *B. oleracea* var. *capitata* (Wang et al., 2012), which can differentiate *OguCMSHY*, *OguCMSR*_1–2_ (420 bp) from *pol* CMS and *OguCMSR*_3_ (471 bp). Similar findings were reported for five CMS lines of broccoli (Shu et al., 2016). Therefore, the original source of *Ogu*CMS in the studied cauliflower CMS lines could have come from *OguCMSR*_1–2_ and *OguCMSHY*. The sequence analysis revealed that SNPs and insertion/deletions could explain the cytoplasmic genetic variations among the CMS lines of Indian cauliflowers. Polymorphic amplicons targeting *orf125* coding regions of mitochondrial genome demonstrated a deletion of 51 nucleotides in the CMS lines with varied floral deformities. Similarly, the deformed CMS lines had a deletion of 31 nucleotides in the coding region of *BnTR4.* Wang et al. (2012) successfully reported the use of chloroplast (cpSSRs) and mitochondrial (mt-SSRs) primers in demonstrating the polymorphism in alloplasmic CMS lines of cabbage. Shu et al. (2015; Shu et al., 2016) demonstrated the role of organelle SSRs and mt-DNA-specific and mt-SSR markers in distinguishing different CMS types, differentiating CMS lines with carpelloid stamens in broccoli. Cytoplasmic genetic variations associated with deletions of 31/51 nucleotides in the *ORF* exonic region demonstrated that insertions/deletions of nucleotides can explain the wide range of floral abnormalities in the CMS lines of Indian cauliflowers. Type and extent of deformities in different CMS lines varied even after the presence of deletions of 31/51 bp, indicating a possible role of nuclear genome, cytonuclear interaction, and environment in determining the type and prevalence of malformities. Therefore, further in-depth studies are needed to pinpoint the exact role of the promoter, coding regions, and epigenetics in determining the specific type and distribution of deformities in the CMS population. However, it was established in the present study that combined use of the primers P15 and P16 can identify the suitable source of male sterile cytoplasm which will provide normal floral structures after their introgression into *B. oleracea* nuclear backgrounds. Therefore, these two identified markers will be extremely useful to the *B. oleracea* geneticists and breeders in developing good CMS lines for use in commercial hybrid breeding and further genetic studies.

Cytonuclear genomic incompatibilities and introgression of alien cytoplasm have been reported to display complex floral structure variations in auto- and alloplasmic CMS lines of *Brassica* crops. The flowering regulation in higher plants is a highly complex process controlled by interactions of genes or genes × environment. MADS-box genes are crucial player in plants for vegetative and reproductive growth development (Meur et al., 2006; Zhang et al., 2011, 2018; Saha et al., 2016; Liu et al., 2018). The identity and development of each floral reproductive whorl is determined by varying combinations of genes of classical flowering ABCDE model (Sheng et al., 2019). It is well documented that loss of function, mutations, or insertions/deletions in MADS-box transcription factors such as *APETALA2*, *APETALA3*, or *PISTILLATA*, *AGAMOUS*, *AGL11*, *SEP1*, *SEP2*, *SEP3*, *SEP4*, *SHP2*, *SHP2*, etc. causes varying degree of floral malformations (Meur et al., 2006; Zhang et al., 2011, 2018; Saha et al., 2016; Liu et al., 2018; Sheng et al., 2019). Different types of floral abnormalities observed in the cauliflower cytolines (Figure 4) such as homeotic-like modification of stamens to petal-like structures (petaloidy), adherence of functional stamens to style, partially opened flowers, splitted style with exposed ovule, splitted style, absence of nectaries, rudimentary nectaries, and fused flowers could be explained by absence of amino acids causing dysfunction of one of MADS-box genes. Furthermore, the dosage imbalance of class B and class C genes of classical flowering model, aberrant mitochondrial gene expression results in homeotic-like floral deformities observed in the present investigation (Meur et al., 2006; Liu et al., 2018). Previously, the role of deletions of nucleotide in the *ORF* coding region of broccoli cytolines was suggested to be associated with carpelloid stamen phenotype (Shu et al., 2015, 2016); however, their role in causing an array of floral deformities is not reported. Identification of these specific deletions associated with several floral deformities would be instrumental in selection of desirable CMS types for hybrid breeding and will enhance the understanding about evolutionary relationships in *Brassicas.* Analysis of *ORFs*, similarity of genes, and *protein* sequence revealed that polymorphic loci were located at exonic regions of *orf125* and *orf108c* of mitochondrial genomes of *Brassicaceae* species. It is quite likely that *orf125* and *orf108c* mitochondrial genes are playing pivotal role in development of flower organs. The floral abnormalities which are associated with aberrant mitochondrial gene expression, mutation in mitochondrial genomes, cytoplasmic-nuclear conflict, cytoplasm types, and genetic backgrounds render scanty nectar production and impaired pollination and affect female fertility (Shu et al., 2015, 2019; Dey et al., 2017b; Kang et al., 2017). These characteristics consequently results in poor seed yield. Identification and roughing out undesirable cytolines in the initial stage of the breeding program will save huge time efforts in developing CMS lines for their successful use in hybrid development.

In the present investigation and our earlier reports (Dey et al., 2011b, 2017b), we had observed floral abnormalities in CMS lines in the successive nuclear backgrounds. Initially, the expression of floral deformities may be weak or incomplete because of partial substitution of nuclear genome. The exact expressions were observed only after complete nuclear substitution. The identification of specific insertion/deletion and SNPs associated with floral abnormalities in the initial stage of backcross introgression will be extremely helpful to the breeders to use only those CMS types which will later results in normal flower structures without any deformities. The proclivity of plant mitochondrial genome to frequent recombination, genomic rearrangements, and rapid structural evolution leading to heteroplasmy results in polymorphism in cauliflower cytoplasmic sources (McCauley, 2013; Yang et al., 2018). Predominantly, the uniparental, which is a maternal inheritance of chloroplast and mitochondrial genome, is reported in most of the angiosperms (Birky, 1995), including *Brassica* crops such as broccoli, cabbage, and cauliflower (Reboud and Zeyl, 1994; Zhang et al., 2012; Shu et al., 2015). Recently, the paternal (Erickson and Kemble, 1990; McCauley, 2013; Worth et al., 2014) and biparental (Hansen et al., 2007; Weihe et al., 2009) inheritance of mitogenome is also reported. Therefore, the findings of the present study pave the way for further analysis to detect any possible paternal inheritance of mitochondrial genome.

Pollinators play an important role in hybrid breeding of *Brassicas*, and pollinator visit is determined by flower phenotype like size, color, and form (Shu et al., 2019). Usually, the large flower size is positively linked with more numbers of pollinator visits, as large floral organ size is generally assumed to contain more nectar (Fenstter et al., 2006; Gomez et al., 2008; Parachnowitsch et al., 2019; Shu et al., 2019). Floral nectar is the primary reward besides pollen, floral oils, resins, and scents, offered to pollinators, and their proportionate ratio is vital in determining plant-pollinator interactions (Gomez et al., 2008). Floral organ size, morphology, proper development of nectaries, efficient nectar production, fruiting features, and pollinator attracting ability are considered potent indexes to evaluate cytolines (Shu et al., 2019). The cytoplasmic effects on floral phenotypes are well documented in *Brassicas* which are manifested through cytonuclear allelic interactions. The role of additive mitonuclear genetic effects in altering floral phenotypes is rare, while epistatic cytonuclear genetic effects are larger in magnitude (Dobler et al., 2014; Roux et al., 2016). In the present investigation, the effects of cytonuclear interactions on floral qualitative traits were insignificant in different nuclear backgrounds (Supplementary Table 3 and Supplementary Figures 2, 3). However, varying degree of cytoplasmic effects on floral reproductive whorls, floral size, structure, and other floral-nectar phenotypes were observed over the successive backcross generations in the development of cauliflower CMS lines (Supplementary Table 4). The introgression of sterile cytoplasm significantly reduced the flower size in the form of reduction in petal length, petal width, and sepal length across the nuclear background series. The other predominant types of structural changes in reproductive whorls were flowers devoid of nonfunctional stamens, alteration in style length, reduction in functional stamens length, modification in position of style and anthers (style position was higher than anthers across the nuclear background series with a few exceptions), and inefficient nectar production. The genetic conflict of sterile cytoplasm-nuclear genome of *B. oleracea* var. *botrytis* could explain the floral deformities (Chen et al., 2017; Singh et al., 2019a). In *Arabidopsis*, the role of cytonuclear epistasis on various adaptive traits is suggested (Roux et al., 2016). Singh and Srivastava (2006) observed the narrowing of petals, reduction of filament length, and increase in style length in the *B. juncea* male sterile lines with the introgression of *ogura*, *tournefortii*, *trachystoma*, and *siifolia* wild cytoplasm. They also obtained the erratic results with the *ogura* cytoplasm in the different nuclear backgrounds because of cytonuclear epistasis. Although, it is also suggested that rigorous recurrent manual or honey bee-mediated selection during backcross breeding cycles could reduce floral deformities to a limited extent (Dey et al., 2017b). However, it is not possible to ameliorate these deformities through conventional backcrossing and different selection strategies.

Understanding the molecular mechanisms and pathways of CMS in cauliflower associated with floral abnormalities along with determining the paternal leakage (if any) need further in depth investigation. Identified molecular markers associated with different kinds of floral deformities will facilitate the selection of CMS lines with normal flower structure at the initial stage of breeding. The establishment of InDel-associated cytoplasmic genetic variations and their association with floral deformities provides reference for understanding developmental biology of floral organ development in CMS lines. The results will also pave the way for elaborating the role of cytonuclear interactions on CMS phenotypes in crop plants.

## Data Availability Statement

The datasets presented in this study can be found in online repositories. The names of the repository/repositories and accession number(s) can be found in the article/Supplementary Material.

## Author Contributions

SD and RB conceived and designed the study. SS and SD performed the experiments. SS did the data recording and lab experiments with input from KS, HG, and KK. SS analyzed the data. AP helped in recording of data related to the reproductive structure of the CMS lines. RK, SD, and RB monitored and contributed the materials. SS performed the sequencing and other molecular analysis. SS wrote the original draft of the manuscript. SD, TB, RK, and RCB edited the final manuscript. All the authors read and approved the final manuscript. All authors contributed to the article and approved the submitted version.

## Conflict of Interest

The authors declare that the research was conducted in the absence of any commercial or financial relationships that could be construed as a potential conflict of interest.

## Footnotes

1 https://blast.ncbi.nlm.nih.gov/

2 https://www.dnastar.com/

3 https://www.ncbi.nlm.nih.gov/orffinder/

## Supplementary Material

Supplementary Figure 1 Principal component analysis and neighbor-joining cluster analysis. The cluster analysis of cauliflower cytolines of varying nuclear genome background based on combined analysis of mt-Dna-specific and mt-Ssr primers is presented here. **(a)** Principal component analysis of 76 cytolines based on molecular data. **(b)** Nj dendrogram of cytolines depicting six distinct groups in different colors.

Supplementary Figure 2 Floral structure of cauliflower cytolines in different nuclear backgrounds.

Supplementary Figure 3 Comparative flower morphology of cytolines and their male fertile counterparts. The upper lane in each image represents male fertile maintainer lines and respective cytolines are in lower lane.

Supplementary Table 1 List of mitochondrial primers used in the present investigation.

Supplementary Table 2 Sequence identity and gene similarity analysis of polymorphic amplicons of cauliflower cytolines targeting *orf125* location with corresponding sequences of *Brassicaceae* mitochondrial genomes.

Supplementary Table 3 Impact of cytonuclear interactions on floral qualitative traits of cauliflower cytolines in different nuclear backgrounds.

Supplementary Table 4 Impact of cytonuclear interactions on floral reproductive traits of cauliflower cytolines in different nuclear backgrounds.
