# Supplementary figures and images for: Elucidating Mitochondrial DNA Markers of Ogura-Based CMS Lines in Indian Cauliflowers (Brassica oleracea var. botrytis L.) and Their Floral Abnormalities Due to Diversity in Cytonuclear Interactions
Source: Front Plant Sci. 2021 Apr 30;12:631489. doi: 10.3389/fpls.2021.631489 (PMC8120243; doi:10.3389/fpls.2021.631489)

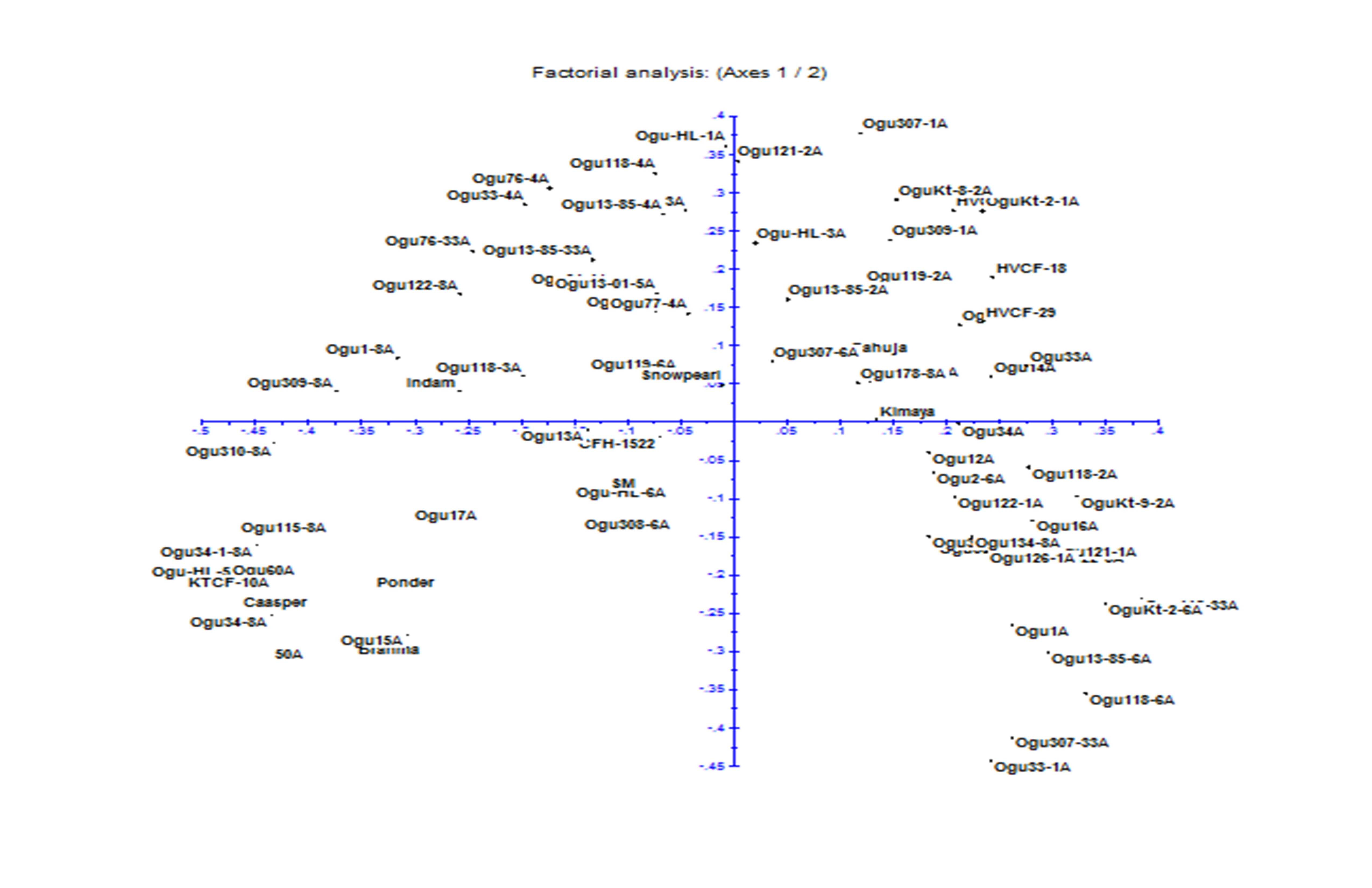

Supplement: Supplementary Figure 1 — Principal component analysis and neighbor-joining cluster analysis. The cluster analysis of cauliflower cytolines of varying nuclear genome background based on combined analysis of mt-Dna-specific and mt-Ssr primers is presented here. (a) Principal component analysis of 76 cytolines based on molecular data. (b) Nj dendrogram of cytolines depicting six distinct groups in different colors. [file Image_1.TIF]

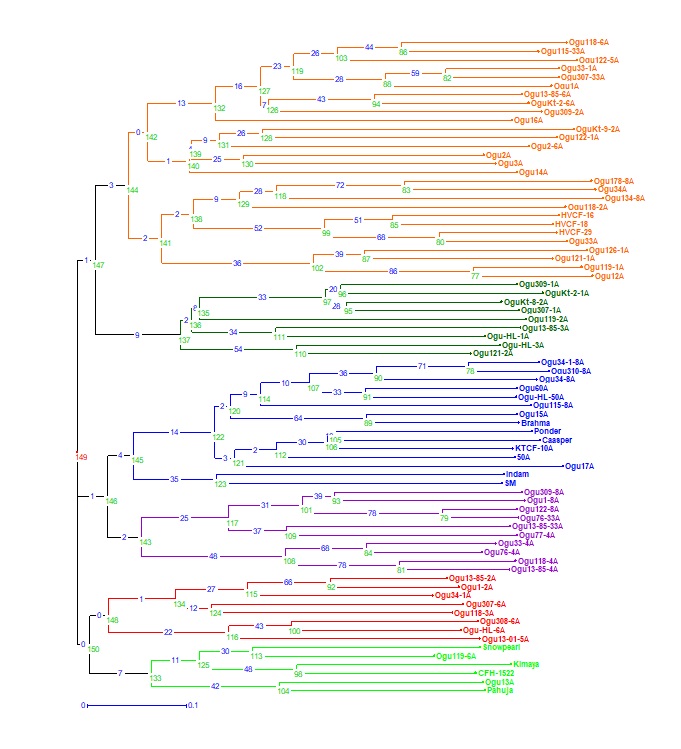

Supplement: Supplementary Figure 2 — Floral structure of cauliflower cytolines in different nuclear backgrounds. [file Image_2.JPEG]

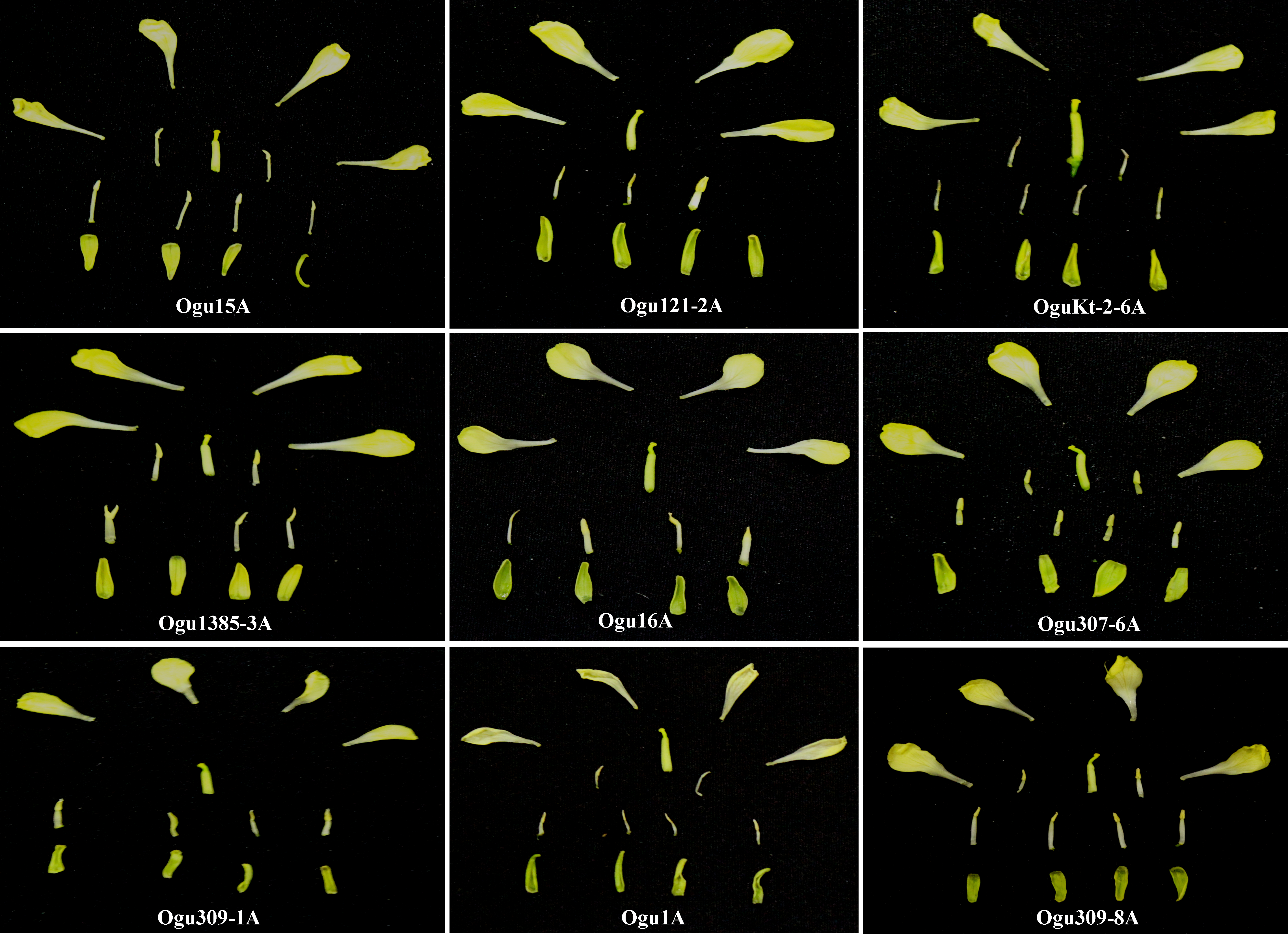

Supplement: Supplementary Figure 3 — Comparative flower morphology of cytolines and their male fertile counterparts. The upper lane in each image represents male fertile maintainer lines and respective cytolines are in lower lane. [file Image_3.TIF]

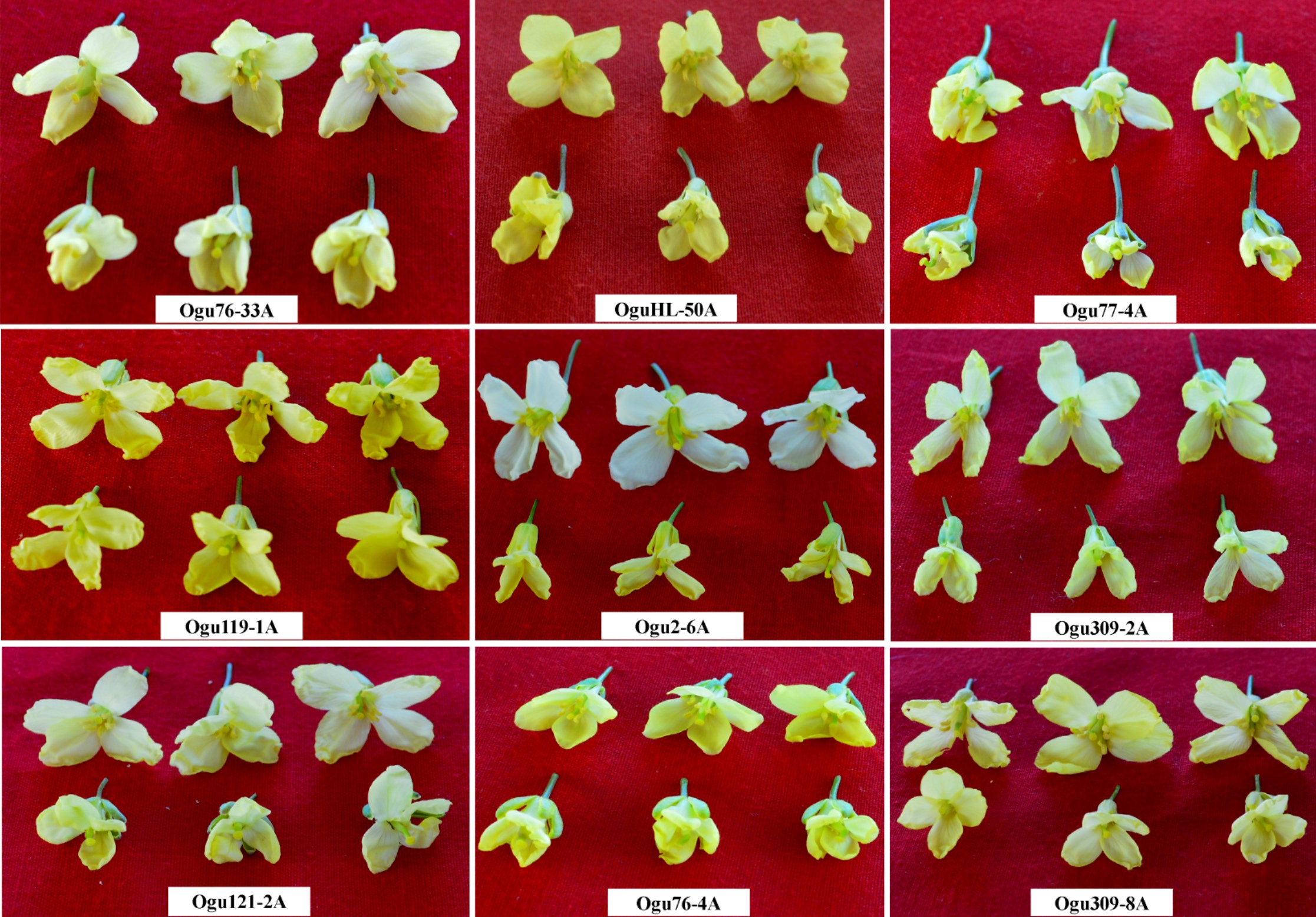

Supplement: Supplementary file 4 [file Image_4.JPEG]
