# Supplementary material for: Elucidating Mitochondrial DNA Markers of Ogura-Based CMS Lines in Indian Cauliflowers (Brassica oleracea var. botrytis L.) and Their Floral Abnormalities Due to Diversity in Cytonuclear Interactions
Source: Front Plant Sci. 2021 Apr 30;12:631489. doi: 10.3389/fpls.2021.631489 (PMC8120243; doi:10.3389/fpls.2021.631489)
Supplement: Supplementary Table 1 — List of mitochondrial primers used in the present investigation. [file Data_Sheet_1.docx]

**Supplementary Table 1.** List of mitochondrial primers used in the present investigation (Shu et al., 2016)

| **Name** | **Sequence** | **Annealing temperature (°C)** | **Target/Locations** |
| --- | --- | --- | --- |
| P1F | GAAACGGGAAGTGACAAT | 54 | *orf138* |
| P1R | GCATTATTTTCTCGGTCCAT |  |  |
| P2F | AGCTGTCTGGAGGGAATC | 54 | *orf222* |
| P2R | GCGGTCTCACGCACTAATC |  |  |
| P3F | ATGCCTCAACTGGATAAAT | 55 | *orf222* |
| P3R | TCATCGAAATAGATCGAGTA |  |  |
| P4F | GCCTCAACTGGATAAATTC | 54 | *orf222-orf224* |
| P4R | CAAGGATCTCGTTCACCT |  |  |
| P5F | AGCTGTCTGGAGGGAATC | 55 | *orf224* |
| P5R | ACGACATCAAGGAGGAAC |  |  |
| P6F | TGAAATGGGAGGTCAGAAGC | 56 | *atp6-orf224* |
| P6R | AAAAGGTGCTAACGGCAGTG |  |  |
| P7F | ATGAAAAATAGACTCCAA | 56 | *orf263* |
| P7R | TCAGTCTAGATAATGCCG |  |  |
| P8F | GCAATGATTACCTTTTTCGA | 55 | *orf138* |
| P8R | GCATTATTTTCTCGGTCCAT |  |  |
| P9F | GAAACGGGAAGTGACAATA | 55 | *orf138* |
| P9R | GCATTATTTTCTCGGTCCAT |  |  |
| P10F | CCATATTTGGCTAAGCTGGTTTTCT | 57 | *orf138* |
| P10R | TATTTTCTCGGTCCATTTTCCAC |  |  |
| P11F | GCCCATATTTGGCTAAGCTG | 56 | *orf138* |
| P11R | TTTTCTCGGTCCATTTTCCA |  |  |
| P12F | CGGTCGGTGTCCAAGATTT | 58 | *orf138* |
| P12R | ACTGTTGGGGTCCTTGCTCT |  |  |
| P13F | AATGAAGCTGTCTGGAGGGA | 56 | *orf138* |
| P13R | TTCATTGAATACTTCCATACCTG |  |  |
| P14F | CCGTTAGGGGTATTTAGTAACTCG | 56 | *BnTR1* |
| P14R | ACATAATGGCAATGTATCGGACTG |  |  |
| P15F | GAAGTCCGAGGACCTTTAGTACC | 56 | *BnTR4* |
| P15R | AGTAAGTTGTAGGTAGGGGCTTCAT |  |  |
| P16F | ACCAAGATTGAGCCAGAT | 55 | *orf125* |
| P16R | CGTCCACTACCGAAAGAG |  |  |
| P17F | CCCGAGAAGCACTGTTGA | 55 | *trnY-trnD* |
| P17R | ACGGAGTGACAAAGGAGC |  |  |
| P18F | CCTTCTGGGTTGACTTGA | 55 | *nad4L-orf101b* |
| P18R | AGTGGTGCCCTCCTCTAA |  |  |
| P19F | GCTGCTCATCACTACCTG | 55 | *orf448-nad6* |
| P19R | CACTACGCTCACTGAAACTA |  |  |

Source: Shu et al., 2016

**Supplementary Table 2.** Sequence identity and gene similarity analysis of polymorphic amplicons of cauliflower cytolines targeting *orf125* location with corresponding sequences of *Brassicaceae* mitochondrial genomes

| **Accession** | **Sequence Identity** | **Protein Name** | **Sequence Identity** | **Protein Name** |
| --- | --- | --- | --- | --- |
|  | YP_004927594.1  (*Brassica carinata*) |  | YP_004927450.1  (*Brassica oleracea,* wild cabbage) |  |
| MN549527 | 95.83% | orf108c | 77.53% | orf125 |
| MN549528 | 83.33% | orf108c | 67.42% | orf125 |
| MN549529 | 82.43% | orf108c | 67.03% | orf125 |
| MN549531 | 72.37% | orf108c | 94.74% | orf125 |
| KU831325.1 | 78.48% | orf108c | 100.00% | orf125 |
| AB694744.1 | 100.00% | orf108c | 77.33% | orf125 |
| AP012990.1 | 100.00% | orf108c | 77.33% | orf125 |

**Supplementary Table 3.** Impact of cytonuclear interactions on floral qualitative traits of cauliflower cytolines in different nuclear backgrounds

| **CMS vs Maintainers** | **Petal Color** | **Shape of style** | **Presence of floral nectaries** | **Presence of viable pollen** | **Type of ovary** |
| --- | --- | --- | --- | --- | --- |
| Ogu33-1A | White | Straight to slightly curved | Present | Absent | Normal |
| Kt-33B1 | Whitish | Straight to slightly curved | Present | Present | Normal |
| Ogu33A | White | Straight to slightly curved | Present | Absent | Normal |
| Kt-33B | Whitish | Straight to slightly curved | Present | Present | Normal |
| Ogu34-1A | White | Straight to slightly curved | Present | Absent | Normal |
| Kt-34B (WF) | White | Straight to slightly curved | Present | Present | Normal |
| Ogu34A | Yellow | Straight to slightly curved | Present | Absent | Normal |
| Kt-34B (YF) | Yellow | Straight to slightly curved | Present | Present | Normal |
| Ogu15A | Yellow | Straight to slightly curved | Present | Absent | Normal |
| Kt-15B | Yellow | Straight to slightly curved | Present | Present | Normal |
| Ogu17A | Whitish Yellow | Straight to curved | Present | Absent | Normal |
| Kt-17B | Whitish Yellow | Straight to slightly curved | Present | Present | Normal |
| Ogu119-1A | Yellow | Straight to slightly curved | Present | Absent | Normal |
| RSK-119B | Yellow | Straight to slightly curved | Present | Present | Normal |
| Ogu307-1A | Yellow | Straight to curved | Present | Absent | Normal |
| Kt-307B | Yellow | Straight to curved | Present | Present | Normal |
| Ogu309-1A | Yellow | Straight to curved | Present | Absent | Normal |
| Kt-309B | Yellow | Straight | Present | Present | Normal |
| Ogu-HL-1A | Yellow | Straight to curved | Present | Absent | Normal |
| HL | Yellow | Straight to slightly curved | Present | Present | Normal |
| Ogu309-2A | Yellow | Straight to curved | Present | Absent | Normal |
| Ogu309B | Yellow | Straight | Present | Present | Normal |
| Ogu1301-5A | White | Straight to curved | Present | Absent | Normal |
| Kt-1301B | Whitish Yellow | Straight to curved | Present | Present | Normal |
| Ogu-HL-3A | Yellow | Straight to curved | Present | Absent | Normal |
| HL | Yellow | Straight to slightly curved | Present | Present | Normal |
| Ogu76-4A | Yellow | Straight to slightly curved | Present | Absent | Normal |
| DC-76 | Yellow | Straight to slightly curved | Present | Present | Normal |
| Ogu76-33A | Yellow | Straight to slightly curved | Present | Absent | Normal |
| DC-76 | Yellow | Straight to slightly curved | Present | Present | Normal |
| Ogu77-4A | Yellow | Straight to curved | Present | Absent | Normal |
| Kt-77B | Whitish Yellow | Straight to slightly curved | Present | Present | Normal |
| Ogu310-8A | Yellow | Straight to slightly curved | Present | Absent | Normal |
| Kt-310B | Yellow | Straight to curved | Present | Present | Normal |
| Ogu178-8A | Yellow | Straight to curved | Present | Absent | Normal |
| Kt-178B | Yellow | Straight to slightly curved | Present | Present | Normal |
| Ogu-HL-50A | Yellow | Straight to curved | Present | Absent | Normal |
| HL | Yellow | Straight to slightly curved | Present | Present | Normal |
| Ogu13-85-4A | Yellow | Straight to slightly curved | Present | Absent | Normal |
| Kt-1385B | Yellow | Straight to slightly curved | Present | Present | Normal |
| Ogu134-8A | White | Straight to slightly curved | Present | Absent | Normal |
| Kt-34B (WF) | White | Straight to slightly curved | Present | Present | Normal |

**Supplementary Table 3. (Continue)**

| **CMS vs Maintainers** | **Petal Color** | **Shape of style** | **Presence of floral nectaries** | **Presence of viable pollen** | **Type of ovary** |
| --- | --- | --- | --- | --- | --- |
| Ogu1A | Whitish Yellow | Straight to slightly curved | Present | Absent | Normal |
| Kt-1B | Whitish Yellow | Straight to slightly curved | Present | Present | Normal |
| Ogu12A | Yellow | Straight to slightly curved | Present | Absent | Normal |
| Kt-12B | Yellow | Straight to slightly curved | Present | Present | Normal |
| Ogu16A | Whitish Yellow | Straight to curved | Present | Absent | Normal |
| Kt-16B | Whitish Yellow | Straight to slightly curved | Present | Present | Normal |
| OguKt-2-1A | Yellow | Straight to curved | Present | Absent | Normal |
| Kt-2B | Yellow | Straight to slightly curved | Present | Present | Normal |
| Ogu121-1A | Yellow | Straight to curved | Present | Absent | Normal |
| Kt-121B | Yellow | Straight to slightly curved | Present | Present | Normal |
| Ogu121-2A | Yellow | Straight to slightly curved | Present | Absent | Normal |
| Kt-121B | Yellow | Straight to slightly curved | Present | Present | Normal |
| Ogu1-2A | Yellow | Straight to slightly curved | Present | Absent | Normal |
| Kt-2B | Yellow | Straight to slightly curved | Present | Present | Normal |
| CB-1 x PHJ | Whitish Yellow | Straight to curved | Present | Absent | Normal |
| PHJ | Whitish Yellow | Straight to slightly curved | Present | Present | Normal |
| Kn81 x HL | Whitish Yellow | Straight to slightly curved | Present | Absent | Normal |
| HL | Yellow | Straight to slightly curved | Present | Present | Normal |
| Ogu122-5A | Whitish Yellow | Straight to slightly curved | Present | Absent | Normal |
| Kt-22B | Yellow | Straight to slightly curved | Present | Present | Normal |
| Ogu122-8A | Whitish Yellow | Straight to slightly curved | Present | Absent | Normal |
| Kt-22B | Yellow | Straight to slightly curved | Present | Present | Normal |
| Ogu309-8A | Yellow | Straight to slightly curved | Present | Absent | Normal |
| Kt-309-8B | Yellow | Straight to slightly curved | Present | Present | Normal |
| Ogu118-4A | Whitish Yellow | Straight to slightly curved | Present | Absent | Normal |
| Kt-18B | Whitish | Straight to slightly curved | Present | Present | Normal |
| Kn81.1301 | Whitish | Straight to curved | Present | Absent | Normal |
| Kt-1301B | Whitish Yellow | Straight to curved | Present | Present | Normal |
| Ogu13-85-33A | Yellow | Straight to curved | Present | Absent | Normal |
| Kt-1385B | Yellow | Straight to slightly curved | Present | Present | Normal |
| Ogu122-1A | Yellow | Straight to curved | Present | Absent | Normal |
| Kt-22B | Yellow | Straight to slightly curved | Present | Present | Normal |
| Ogu126-1A | Yellow | Straight to slightly curved | Present | Absent | Normal |
| Kt-126B | Yellow | Straight to slightly curved | Present | Present | Normal |
| OguKt-9-2A | Whitish Yellow | Straight to slightly curved | Present | Absent | Normal |
| Kt-9B | Whitish Yellow | Straight to slightly curved | Present | Present | Normal |
| OguKt-8-2A | Whitish Yellow | Straight to slightly curved | Present | Absent | Normal |
| Kt-8B | Whitish Yellow | Straight to slightly curved | Present | Present | Normal |
| Ogu118-3A | Whitish Yellow | Straight to scurved | Present | Absent | Normal |
| Kt-18B | Whitish | Straight to slightly curved | Present | Present | Normal |
| Ogu308-6A | Yellow | Straight to slightly curved | Present | Absent | Normal |
| Kt-308B | Yellow | Straight to slightly curved | Present | Present | Normal |

**Supplementary Table 3. (Continue)**

| **CMS vs Maintainers** | **Petal Color** | **Shape of style** | **Presence of floral nectaries** | **Presence of viable pollen** | **Type of ovary** |
| --- | --- | --- | --- | --- | --- |
| Ogu2-6A | Whitish Yellow | Straight to slightly curved | Present | Absent | Normal |
| Kt-1B | Whitish Yellow | Straight to slightly curved | Present | Present | Normal |
| Ogu115-33A | Whitish Yellow | Straight to slightly curved | Present | Absent | Normal |
| Kt-15B | Whitish Yellow | Straight to slightly curved | Present | Present | Normal |
| Ogu307-33A | Yellow | Straight to slightly curved | Present | Absent | Normal |
| Kt-307-33B | Yellow | Straight to slightly curved | Present | Present | Normal |
| Ogu1-8A | Whitish Yellow | Straight to slightly curved | Present | Absent | Normal |
| Kt-1B | Whitish Yellow | Straight to slightly curved | Present | Present | Normal |
| Ogu13-85-3A | Yellow | Straight to slightly curved | Present | Absent | Normal |
| Kt-1385B | Yellow | Straight to slightly curved | Present | Present | Normal |
| Ogu2A | Yellow | Straight to slightly curved | Present | Absent | Normal |
| Kt-2B | Whitish Yellow | Straight to slightly curved | Present | Present | Normal |
| Ogu3A | Yellow | Straight to slightly curved | Present | Absent | Normal |
| Kt-3B | Yellow | Straight to curved | Present | Present | Normal |
| Ogu13A | White | Straight to curved | Present | Absent | Normal |
| Kt-13B | White | Straight to slightly curved | Present | Present | Normal |
| Ogu14A | Yellow | Straight to slightly curved | Present | Absent | Normal |
| Kt-14B | Yellow | Straight to slightly curved | Present | Present | Normal |
| Ogu119-2A | Yellow | Straight to slightly curved | Present | Absent | Normal |
| RSK-119B | Yellow | Straight to slightly curved | Present | Present | Normal |
| Ogu13-85-2A | Yellow | Straight to slightly curved | Present | Absent | Normal |
| Kt-1385B | Yellow | Straight to slightly curved | Present | Present | Normal |
| Ogu118-2A | White | Straight to slightly curved | Present | Absent | Normal |
| Kt-18B | White | Straight to slightly curved | Present | Present | Normal |
| Ogu119-6A | Yellow | Straight to slightly curved | Present | Absent | Normal |
| RSK-119B | Yellow | Straight to slightly curved | Present | Present | Normal |
| Ogu13-85-6A | Yellow | Straight to slightly curved | Present | Absent | Normal |
| Kt-1385B | Yellow | Straight to slightly curved | Present | Present | Normal |
| Ogu118-6A | White | Straight to slightly curved | Present | Absent | Normal |
| Kt-18B | White | Straight to slightly curved | Present | Present | Normal |
| OguKt-2-6A | White | Straight to curved | Present | Absent | Normal |
| Kt-2B | Whitish | Straight to slightly curved | Present | Present | Normal |
| 33A x1301 | Whitish | Straight to curved | Present | Absent | Normal |
| Kt-1301B | Whitish Yellow | Straight to curved | Present | Present | Normal |
| Ogu115-8A | Whitish | Straight to curved | Present | Absent | Normal |
| Kt-15B | Whitish Yellow | Straight to slightly curved | Present | Present | Normal |

**Supplementary Table 4.** Impact of cytonuclear interactions on floral reproductive traits of cauliflower cytolines in different nuclear backgrounds

| **CMS vs Maintainers** | **Petal Size (mm)** | |  | **Sepal Size (mm)** | |  | **Filament length (mm)** | | **Stamen length (mm)** | |  |  |  |
| --- | --- | --- | --- | --- | --- | --- | --- | --- | --- | --- | --- | --- | --- |
|  | **Petal Length** | **Petal Width** | **L:W** | **Sepal Length** | **Sepal Width** | **SL:SW** | **Short** | **Long** | **Short** | **Long** | **Style Length (mm)** | **Stamen:Style** | **Stamen L: Stamen S** |
| Ogu33-1A | 15.70** | 6.18** | 2.55** | 7.51 | 3.16 | 2.40 | 4.28** | 5.74** | 5.84** | 7.60** | 6.98 | 1.09** | 1.34 |
| Kt-33B1 | 18.08 | 8.29 | 2.19 | 7.63 | 3.09 | 2.48 | 6.28 | 8.02 | 8.27 | 9.61 | 6.86 | 1.41 | 1.16 |
| Ogu33A | 15.92 | 6.54 | 2.46 | 7.60** | 3.11 | 2.45* | 2.74** | 4.68** | 4.03** | 7.12* | 6.54 | 1.09** | 1.80** |
| Kt-33B | 16.57 | 6.38 | 2.60 | 6.70 | 3.14 | 2.15 | 5.21 | 6.39 | 6.41 | 8.32 | 6.33 | 1.32 | 1.30 |
| Ogu34-1A | 15.35** | 5.48** | 2.81** | 7.11** | 3.13* | 2.28 | 3.53** | 5.96 | 5.63 | 7.73 | 8.68** | 0.90** | 1.39* |
| Kt-34B (WF) | 17.00 | 8.18 | 2.09 | 7.68 | 3.29 | 2.34 | 5.08 | 6.59 | 6.50 | 8.03 | 7.02 | 1.15 | 1.25 |
| Ogu34A | 15.70** | 6.22** | 2.53 | 6.93* | 2.91* | 2.40 | 3.04** | 5.95** | 4.38** | 7.52** | 6.78 | 1.11* | 1.80** |
| Kt-34B (YF) | 16.78 | 6.93 | 2.43 | 7.24 | 3.14 | 2.31 | 6.18 | 7.34 | 7.89 | 8.99 | 6.96 | 1.30 | 1.14 |
| Ogu15A | 13.66** | 4.70** | 2.93** | 6.23** | 2.88* | 2.19 | 2.64** | 3.98** | 3.99** | 5.80** | 6.56** | 0.88** | 1.46** |
| Kt-15B | 16.83 | 6.28 | 2.68 | 7.05 | 3.07 | 2.30 | 6.85 | 8.49 | 8.16 | 10.28 | 7.64 | 1.35 | 1.26 |
| Ogu17A | 13.23** | 5.27** | 2.53** | 7.45** | 3.24* | 2.30** | 3.05** | 5.73** | 4.97** | 7.67** | 7.84** | 0.98** | 1.57** |
| Kt-17B | 16.87 | 8.60 | 1.97 | 8.39 | 2.89 | 2.95 | 8.32 | 9.59 | 9.84 | 10.99 | 8.71 | 1.26 | 1.12 |
| Ogu119-1A | 14.58** | 5.86** | 2.49* | 8.12** | 3.12 | 2.63** | 3.04** | 4.34** | 4.49** | 6.45** | 6.83** | 0.94** | 1.45** |
| RSK-119B | 19.31 | 8.18 | 2.36 | 9.98 | 3.19 | 3.13 | 7.11 | 8.40 | 8.64 | 10.09 | 8.22 | 1.23 | 1.17 |
| Ogu307-1A | 13.23** | 6.68** | 1.99 | 6.53** | 2.64** | 2.50 | 3.98** | 5.01** | 5.89** | 7.25** | 6.67** | 1.09** | 1.23* |
| Kt-307B | 16.66 | 8.43 | 1.98 | 8.55 | 3.27 | 2.62 | 7.43 | 8.73 | 8.93 | 10.30 | 7.58 | 1.36 | 1.15 |
| Ogu309-1A | 13.16** | 4.87** | 2.73** | 6.37** | 2.58** | 2.49* | 3.13** | 5.03** | 4.53** | 6.61** | 6.33** | 1.05** | 1.48** |
| Kt-309B | 16.47 | 5.38 | 3.06 | 7.38 | 3.24 | 2.28 | 6.25 | 7.28 | 7.31 | 9.00 | 7.39 | 1.22 | 1.23 |
| Ogu-HL-1A | 14.49** | 6.01** | 2.43* | 8.27** | 2.44** | 3.42** | 3.34** | 5.42** | 5.74** | 7.72** | 8.11** | 0.95** | 1.35** |
| HL | 18.19 | 8.10 | 2.25 | 9.58 | 3.30 | 2.91 | 6.82 | 8.73 | 8.33 | 9.94 | 9.32 | 1.07 | 1.19 |
| Ogu309-2A | 14.41** | 5.04 | 2.91 | 6.63** | 2.37** | 2.82** | 3.00** | 4.36** | 4.14** | 6.56** | 6.32** | 1.04** | 1.59** |
| Kt-309B | 16.47 | 5.38 | 3.06 | 7.38 | 3.24 | 2.28 | 6.25 | 7.28 | 7.31 | 9.00 | 7.39 | 1.22 | 1.23 |
| Ogu1301-5A | 14.38** | 6.68** | 2.15** | 7.32** | 3.23 | 2.28** | 3.16** | 4.38** | 5.28** | 6.69** | 8.23** | 0.82** | 1.27** |
| Kt-1301B | 19.81 | 8.33 | 2.38 | 9.74 | 2.99 | 3.28 | 8.20 | 9.78 | 10.00 | 10.96 | 10.48 | 1.05 | 1.10 |

**Supplementary Table 4 (Continue)**

| **CMS vs Maintainers** | **Petal Size (mm)** | |  | **Sepal Size (mm)** | |  | **Filament length (mm)** | | **Stamen length (mm)** | |  |  |  |
| --- | --- | --- | --- | --- | --- | --- | --- | --- | --- | --- | --- | --- | --- |
|  | **Petal Length** | **Petal Width** | **L:W** | **Sepal Length** | **Sepal Width** | **SL:SW** | **Short** | **Long** | **Short** | **Long** | **Style Length (mm)** | **Stamen:Style** | **Stamen L: Stamen S** |
| Ogu-HL-3A | 13.83** | 6.62** | 2.09** | 8.27** | 3.31 | 2.51** | 3.38** | 5.34** | 5.20** | 6.58** | 7.29** | 0.90** | 1.27 |
| HL | 18.19 | 8.10 | 2.25 | 9.58 | 3.30 | 2.91 | 6.82 | 8.73 | 8.33 | 9.94 | 9.32 | 1.07 | 1.19 |
| Ogu76-4A | 12.91** | 5.53** | 2.35** | 7.01** | 2.46** | 2.86* | 3.40** | 4.53** | 4.51** | 6.61** | 6.60** | 1.00** | 1.47 |
| DC-76 | 17.13 | 8.27 | 2.07 | 8.57 | 3.23 | 2.65 | 7.88 | 8.58 | 8.48 | 9.58 | 8.59 | 1.11 | 1.13 |
| Ogu76-33A | 13.83** | 6.62** | 2.09 | 8.27 | 3.31 | 2.51 | 3.38** | 5.34** | 5.20** | 6.58** | 7.29** | 0.90** | 1.27** |
| DC-76 | 17.13 | 8.27 | 2.07 | 8.57 | 3.23 | 2.65 | 7.88 | 8.58 | 8.48 | 9.58 | 8.59 | 1.11 | 1.13 |
| Ogu77-4A | 13.84** | 5.36** | 2.59** | 8.23** | 2.94** | 2.82* | 2.69** | 5.74** | 4.08** | 8.42** | 7.06 | 1.19** | 2.07** |
| Kt-77B | 16.05 | 7.85 | 2.05 | 7.84 | 3.16 | 2.49 | 6.18 | 8.38 | 8.57 | 9.97 | 7.38 | 1.36 | 1.16 |
| Ogu310-8A | 16.65 | 5.63 | 3.00 | 8.23 | 2.40 | 3.46 | 6.38 | 7.52** | 8.13 | 9.15** | 8.90 | 1.03 | 1.13 |
| Kt-310B | 17.33 | 5.32 | 3.26 | 8.07 | 2.34 | 3.46 | 6.78 | 8.67 | 7.93 | 10.19 | 8.55 | 1.19 | 1.30 |
| Ogu178-8A | 15.03** | 7.44** | 2.03** | 8.23** | 2.72** | 3.08 | 3.16** | 4.48** | 4.97** | 6.88** | 8.31 | 0.83** | 1.39** |
| Kt-178B | 18.43 | 10.19 | 1.81 | 9.69 | 3.24 | 2.99 | 8.04 | 9.41 | 9.17 | 10.94 | 8.63 | 1.27 | 1.20 |
| Ogu-HL-50A | 15.40** | 5.71** | 2.71** | 9.19* | 3.28 | 2.81 | 4.54** | 6.08** | 6.41** | 8.77** | 8.59** | 1.02 | 1.37** |
| HL | 18.19 | 8.10 | 2.25 | 9.58 | 3.30 | 2.91 | 6.82 | 8.73 | 8.33 | 9.94 | 9.32 | 1.07 | 1.19 |
| Ogu13-85-4A | 14.66** | 5.06** | 2.91** | 7.83** | 2.75** | 2.86** | 2.86** | 3.56** | 3.46** | 5.28** | 7.22 | 0.74** | 1.54** |
| Kt-1385B | 18.01 | 7.00 | 2.57 | 8.80 | 3.19 | 2.76 | 6.70 | 7.47 | 8.20 | 8.89 | 7.08 | 1.26 | 1.08 |
| Ogu134-8A | 14.76** | 5.76** | 2.59** | 8.23** | 3.15* | 2.62** | 3.28** | 4.24** | 4.36** | 5.39** | 6.52** | 0.83** | 1.24 |
| Kt-34B (WF) | 17.00 | 8.18 | 2.09 | 7.68 | 3.29 | 2.34 | 5.08 | 6.59 | 6.50 | 8.03 | 7.02 | 1.15 | 1.25 |
| Ogu1A | 15.25** | 6.46** | 2.37 | 8.70** | 3.27* | 2.67** | 3.12** | 4.86** | 4.98** | 6.75** | 8.43** | 0.80** | 1.36** |
| Kt-1B | 20.08 | 8.50 | 2.36 | 10.75 | 3.63 | 2.97 | 9.12 | 9.81 | 10.57 | 11.63 | 9.99 | 1.17 | 1.10 |
| Ogu12A | 16.03 | 7.10** | 2.26** | 8.24** | 3.19* | 2.58** | 3.73** | 5.13** | 6.10** | 7.20** | 7.76** | 0.93** | 1.18 |
| Kt-12B | 15.40 | 9.46 | 1.63 | 9.94 | 3.30 | 3.02 | 8.90 | 9.49 | 10.14 | 11.44 | 10.59 | 1.08 | 1.13 |

**Supplementary Table 4 (Continue)**

| **CMS vs Maintainers** | **Petal Size (mm)** | |  | **Sepal Size (mm)** | |  | **Filament length (mm)** | | **Stamen length (mm)** | |  |  |  |
| --- | --- | --- | --- | --- | --- | --- | --- | --- | --- | --- | --- | --- | --- |
|  | **Petal Length** | **Petal Width** | **L:W** | **Sepal Length** | **Sepal Width** | **SL:SW** | **Short** | **Long** | **Short** | **Long** | **Style Length (mm)** | **Stamen:Style** | **Stamen L: Stamen S** |
| Ogu16A | 15.67** | 6.38** | 2.46 | 8.95 | 3.23 | 2.77 | 2.30** | 5.67** | 3.41** | 7.50** | 9.44** | 0.79 | 1.65 |
| Kt-16B | 19.07 | 7.61 | 2.51 | 8.66 | 3.27 | 2.65 | 7.23 | 8.87 | 9.28 | 10.58 | 10.86 | 0.98 | 1.14 |
| OguKt-2-1A | 14.73** | 6.50** | 2.28* | 9.05** | 2.34** | 3.88** | 3.34** | 6.09** | 5.96** | 8.88** | 7.23** | 1.23 | 1.03 |
| Kt-2B | 18.65 | 8.65 | 2.16 | 9.73 | 3.18 | 3.08 | 6.83 | 8.25 | 8.33 | 10.13 | 8.33 | 1.22 | 1.22 |
| Ogu121-1A | 14.84** | 5.49** | 2.74* | 9.33** | 3.09 | 3.05 | - | 4.33** | - | 7.03** | 7.10** | 0.99** | - |
| Kt-121B | 19.86 | 9.58 | 2.08 | 10.61 | 3.25 | 3.27 | 7.23 | 8.30 | 8.75 | 10.65 | 7.30 | 1.46 | 1.22 |
| Ogu121-2A | 16.16** | 6.77** | 2.39** | 8.75** | 2.63** | 3.39 | 2.75** | 5.04** | 4.06** | 7.98** | 8.02** | 1.00** | 1.97** |
| Kt-121B | 19.86 | 9.58 | 2.08 | 10.61 | 3.25 | 3.27 | 7.23 | 8.30 | 8.75 | 10.65 | 7.30 | 1.46 | 1.22 |
| Ogu1-2A | 16.56** | 5.28** | 3.14** | 8.33** | 3.13** | 2.67** | 3.57** | 5.18** | 5.78** | 7.05** | 7.31** | 0.97** | 1.23** |
| Kt-2B | 20.22 | 10.70 | 1.89 | 10.88 | 3.41 | 3.20 | 8.35 | 10.26 | 10.31 | 11.45 | 9.53 | 1.20 | 1.11 |
| Ogu77-2A | 13.48** | 6.16** | 2.21* | 8.21* | 2.30** | 3.59** | 3.46** | 4.95** | 6.33** | 7.93** | 8.02** | 0.99** | 1.25** |
| Kt-77B | 16.05 | 7.85 | 2.05 | 7.84 | 3.16 | 2.49 | 6.18 | 8.38 | 8.57 | 9.97 | 7.38 | 1.36 | 1.16 |
| OguHL-5A | 13.21** | 5.24** | 2.52** | 8.31** | 2.78** | 3.04** | 2.63** | 4.34** | 3.77** | 5.62** | 7.78** | 0.72** | 1.49** |
| HL | 18.19 | 8.10 | 2.25 | 9.58 | 3.30 | 2.91 | 6.82 | 8.73 | 8.33 | 9.94 | 9.32 | 1.07 | 1.19 |
| Ogu122-5A | 15.89** | 6.30** | 2.52** | 8.59** | 3.12** | 2.76 | 3.83** | 7.37** | 6.04** | 9.03* | 7.36** | 1.23** | 1.50** |
| Kt-22B | 16.94 | 9.23 | 1.84 | 8.22 | 2.90 | 2.86 | 5.86 | 8.11 | 7.77 | 9.38 | 8.38 | 1.12 | 1.21 |
| Ogu122-8A | 14.93** | 6.18** | 2.42** | 8.43** | 3.02 | 2.81 | 2.80** | 4.68** | 4.28** | 6.41** | 7.13** | 0.90** | 1.51** |
| Kt-22B | 16.94 | 9.23 | 1.84 | 8.22 | 2.90 | 2.86 | 5.86 | 8.11 | 7.77 | 9.38 | 8.38 | 1.12 | 1.21 |
| Ogu309-8A | 15.50** | 6.96** | 2.23** | 9.00** | 3.10 | 2.91 | 2.88** | 4.21** | 4.75** | 7.03** | 8.14 | 0.86** | 1.48** |
| Kt-309-8B | 18.87 | 8.82 | 2.14 | 9.63 | 3.21 | 3.01 | 8.05 | 9.18 | 9.91 | 10.63 | 8.28 | 1.28 | 1.07 |
| Ogu118-4A | 14.38** | 6.13** | 2.35* | 8.23** | 2.90** | 2.86 | 3.86** | 6.95** | 6.81** | 8.83** | 8.14** | 1.09** | 1.30** |
| Kt-18B | 19.02 | 7.43 | 2.56 | 9.04 | 3.33 | 2.74 | 7.85 | 9.08 | 8.96 | 10.65 | 9.07 | 1.18 | 1.19 |
| Kn81.1301 | 12.92** | 6.63** | 1.95** | 7.43** | 2.88 | 2.59** | 3.23** | 4.38** | 5.68** | 6.96** | 7.58** | 0.92** | 1.23** |
| Kt-1301B | 19.81 | 8.33 | 2.38 | 9.74 | 2.99 | 3.28 | 8.20 | 9.78 | 10.00 | 10.96 | 10.48 | 1.05 | 1.10 |

**Supplementary Table 4 (Continue)**

| **CMS vs Maintainers** | **Petal Size (mm)** | |  | **Sepal Size (mm)** | |  | **Filament length (mm)** | | **Stamen length (mm)** | |  |  |  |
| --- | --- | --- | --- | --- | --- | --- | --- | --- | --- | --- | --- | --- | --- |
|  | **Petal Length** | **Petal Width** | **L:W** | **Sepal Length** | **Sepal Width** | **SL:SW** | **Short** | **Long** | **Short** | **Long** | **Style Length (mm)** | **Stamen:Style** | **Stamen L: Stamen S** |
| Ogu13-85-33A | 15.05** | 5.09** | 2.96** | 8.13** | 2.93** | 2.80 | 3.07** | 4.09** | 5.02** | 7.12** | 7.36** | 0.97** | 1.42** |
| Kt-1385B | 18.01 | 7.00 | 2.57 | 8.80 | 3.19 | 2.76 | 6.70 | 7.47 | 8.20 | 8.89 | 7.08 | 1.26 | 1.08 |
| Ogu122-1A | 15.39** | 6.06** | 2.54** | 7.98** | 2.30** | 3.48** | - | 3.91** | - | 5.03** | 7.18** | 0.71** | - |
| Kt-22B | 16.94 | 9.23 | 1.84 | 8.22 | 2.90 | 2.86 | 5.86 | 8.11 | 7.77 | 9.38 | 8.38 | 1.12 | 1.21 |
| Ogu126-1A | 16.74** | 7.10** | 2.36** | 9.24** | 3.22** | 2.88** | - | 4.14** | - | 6.06** | 8.02** | 0.76** | - |
| Kt-126B | 20.03 | 9.87 | 2.03 | 11.03 | 3.55 | 3.12 | 8.23 | 9.31 | 9.97 | 11.35 | 9.10 | 1.25 | 1.14 |
| OguKt-9-2A | 15.62** | 6.92** | 2.26 | 8.93** | 3.15 | 2.85** | - | 4.09** | - | 7.01** | 6.26** | 1.12** | - |
| Kt-9B | 19.14 | 8.55 | 2.24 | 9.88 | 3.14 | 3.15 | 8.52 | 9.51 | 9.99 | 10.89 | 8.23 | 1.32 | 1.09 |
| OguKt-8-2A | 15.21** | 5.94** | 2.56** | 8.97** | 3.05** | 2.95 | - | 4.26** | - | 7.05** | 7.27** | 0.97** | - |
| Kt-8B | 19.12 | 8.68 | 2.21 | 10.05 | 3.28 | 3.07 | 6.96 | 8.64 | 8.97 | 10.93 | 8.64 | 1.27 | 1.22 |
| Ogu118-3A | 13.43** | 5.94** | 2.26** | 7.13** | 2.45** | 2.94 | - | 4.78** | - | 7.18** | 6.29** | 1.14 | - |
| Kt-18B | 19.02 | 7.43 | 2.56 | 9.04 | 3.33 | 2.74 | 7.85 | 9.08 | 8.96 | 10.65 | 9.07 | 1.18 | 1.19 |
| Ogu308-6A | 15.02** | 6.66** | 2.25 | 8.58** | 3.29 | 2.62 | 3.83** | 5.61** | 6.37** | 7.97** | 7.84** | 1.01** | 1.26** |
| Kt-308B | 18.38 | 8.46 | 2.17 | 9.27 | 3.31 | 2.81 | 8.36 | 9.30 | 9.54 | 11.03 | 9.39 | 1.18 | 1.16 |
| Ogu2-6A | 17.92** | 7.83** | 2.29 | 10.28* | 3.38* | 3.05 | 5.65** | 7.37** | 7.67** | 9.48** | 9.33* | 1.01** | 1.24** |
| Kt-1B | 20.08 | 8.50 | 2.36 | 10.75 | 3.63 | 2.97 | 9.12 | 9.81 | 10.57 | 11.63 | 9.99 | 1.17 | 1.10 |
| Ogu115-33A | 16.64** | 6.89** | 2.42** | 10.12* | 3.29** | 3.08 | 3.67** | 6.41** | 5.23** | 8.57** | 8.25** | 1.05 | 1.64** |
| Kt-15B | 17.93 | 8.73 | 2.07 | 10.68 | 3.58 | 2.99 | 7.90 | 9.46 | 9.90 | 11.33 | 9.96 | 1.14 | 1.15 |
| Ogu307-33A | 15.43** | 6.53** | 2.36* | 7.49** | 3.37 | 2.23* | 3.21** | 3.86** | 5.22** | 5.97** | 6.38** | 0.93** | 1.15 |
| Kt-307-33B | 16.66 | 8.43 | 1.98 | 8.55 | 3.27 | 2.62 | 7.43 | 8.73 | 8.93 | 10.30 | 7.58 | 1.36 | 1.15 |
| Ogu1-8A | 16.11** | 6.66** | 2.42 | 9.07** | 3.39** | 2.68** | 3.05** | 5.05** | 4.84** | 7.08** | 8.13** | 0.87** | 1.46** |
| Kt-1B | 20.08 | 8.50 | 2.36 | 10.75 | 3.63 | 2.97 | 9.12 | 9.81 | 10.57 | 11.63 | 9.99 | 1.17 | 1.10 |

**Supplementary Table 4 (Continue)**

| **CMS vs Maintainers** | **Petal Size (mm)** | |  | **Sepal Size (mm)** | |  | **Filament length (mm)** | | **Stamen length (mm)** | |  |  |  |
| --- | --- | --- | --- | --- | --- | --- | --- | --- | --- | --- | --- | --- | --- |
| CMS line | **Petal Length** | **Petal Width** | **L:W** | **Sepal Length** | **Sepal Width** | **SL:SW** | **Short** | **Long** | **Short** | **Long** | **Style Length (mm)** | **Stamen: Style** | **Stamen L: Stamen S** |
| Ogu13-85-3A | 16.22** | 4.93** | 3.30** | 8.84 | 2.33** | 3.83* | 3.80** | 5.65** | 5.95** | 7.88** | 8.11** | 0.97** | 1.33** |
| Kt-1385B | 18.01 | 7.00 | 2.57 | 8.80 | 3.19 | 2.76 | 6.70 | 7.47 | 8.20 | 8.89 | 7.08 | 1.26 | 1.08 |
| Ogu2A | 15.02** | 5.61** | 2.69** | 8.03** | 3.15 | 2.56** | - | 4.14** | - | 6.10** | 6.34** | 0.96** | - |
| Kt-2B | 18.65 | 8.65 | 2.16 | 9.73 | 3.18 | 3.08 | 6.83 | 8.25 | 8.33 | 10.13 | 8.33 | 1.22 | 1.22 |
| Ogu3A | 13.68** | 5.00** | 2.77** | 7.97** | 2.67** | 3.00 | - | 4.90** | - | 7.24** | 5.97** | 1.22** | - |
| Kt-3B | 17.04 | 7.18 | 2.38 | 9.34 | 3.13 | 2.99 | 7.32 | 9.12 | 8.74 | 10.71 | 7.36 | 1.46 | 1.23 |
| Ogu13A | 15.22** | 6.54** | 2.33** | 9.18** | 2.38** | 3.87** | 3.03** | 4.10** | 3.97** | 5.99** | 7.29** | 0.83** | 1.51** |
| Kt-13B | 19.15 | 7.54 | 2.54 | 10.11 | 3.41 | 2.97 | 7.74 | 9.22 | 9.85 | 10.79 | 8.80 | 1.23 | 1.10 |
| Ogu14A | 14.19** | 5.36** | 2.65** | 7.13** | 3.20 | 2.24** | 2.46** | 3.61** | 3.68** | 6.93** | 5.32** | 1.30 | 1.89** |
| Kt-14B | 18.67 | 8.67 | 2.16 | 9.83 | 3.33 | 2.96 | 7.88 | 9.03 | 9.06 | 11.24 | 8.07 | 1.39 | 1.24 |
| Ogu119-2A | 15.93** | 5.99** | 2.66** | 8.13** | 2.44** | 3.34 | 3.78** | 4.21** | 6.01** | 6.77** | 6.65** | 1.02** | 1.13 |
| RSK-119B | 19.31 | 8.18 | 2.36 | 9.98 | 3.19 | 3.13 | 7.11 | 8.40 | 8.64 | 10.09 | 8.22 | 1.23 | 1.17 |
| Ogu13-85-2A | 15.95** | 4.18** | 3.82** | 7.80** | 2.21** | 3.54** | 3.80** | 4.28** | 5.26** | 7.14** | 6.94** | 1.03** | 1.36** |
| Kt-1385B | 18.01 | 7.00 | 2.57 | 8.80 | 3.19 | 2.76 | 6.70 | 7.47 | 8.20 | 8.89 | 7.08 | 1.26 | 1.08 |
| Ogu118-2A | 15.38** | 5.06** | 3.04** | 8.22** | 2.25** | 3.66** | - | 4.03** | - | 5.86** | 6.98** | 0.84** | - |
| Kt-18B | 19.02 | 7.43 | 2.56 | 9.04 | 3.33 | 2.74 | 7.85 | 9.08 | 8.96 | 10.65 | 9.07 | 1.18 | 1.19 |
| Ogu119-6A | 14.36** | 5.18** | 2.78** | 8.31** | 3.43** | 2.43** | 3.15** | 4.27** | 4.05** | 7.60** | 6.22** | 1.22 | 1.88** |
| RSK-119B | 19.31 | 8.18 | 2.36 | 9.98 | 3.19 | 3.13 | 7.11 | 8.40 | 8.64 | 10.09 | 8.22 | 1.23 | 1.17 |
| Ogu13-85-6A | 13.75** | 4.19** | 3.28** | 7.31** | 2.48** | 2.96 | 3.40** | 4.15** | 5.30** | 6.49** | 6.28** | 1.04** | 1.23** |
| Kt-1385B | 18.01 | 7.00 | 2.57 | 8.80 | 3.19 | 2.76 | 6.70 | 7.47 | 8.20 | 8.89 | 7.08 | 1.26 | 1.08 |
| Ogu118-6A | 15.03** | 5.44** | 2.77 | 8.22** | 2.97* | 2.80 | 2.70** | 4.13** | 3.94** | 5.35** | 6.63** | 0.81** | 1.36** |
| Kt-18B | 19.02 | 7.43 | 2.56 | 9.04 | 3.33 | 2.74 | 7.85 | 9.08 | 8.96 | 10.65 | 9.07 | 1.18 | 1.19 |
| OguKt-2-6A | 15.50** | 6.98** | 2.22 | 8.33** | 3.42* | 2.45** | 3.49** | 4.28** | 5.32** | 6.76** | 8.24 | 0.82** | 1.27 |
| Kt-2B | 18.65 | 8.65 | 2.16 | 9.73 | 3.18 | 3.08 | 6.83 | 8.25 | 8.33 | 10.13 | 8.33 | 1.22 | 1.22 |
| Ogu13-01-33A | 13.49** | 7.19** | 1.88** | 7.27** | 2.83 | 2.58* | 2.84** | 3.40** | 3.64** | 4.79** | 7.69** | 0.62* | 1.32** |
| Kt-1301B | 19.81 | 8.33 | 2.38 | 9.74 | 2.99 | 3.28 | 8.20 | 9.78 | 10.00 | 10.96 | 10.48 | 1.05 | 1.10 |
| Ogu115-8A | 16.26** | 6.58** | 2.47* | 9.82** | 3.53 | 2.79 | 3.24** | 4.10** | 5.08** | 6.63** | 8.18** | 0.81* | 1.31** |
| Kt-15B | 17.93 | 8.73 | 2.07 | 10.68 | 3.58 | 2.99 | 7.90 | 9.46 | 9.90 | 11.33 | 9.96 | 1.14 | 1.15 |
